# Varying Dose of Atropine in Slowing Myopia Progression in Children Over Different Follow-Up Periods by Meta-Analysis

**DOI:** 10.3389/fmed.2021.756398

**Published:** 2022-01-13

**Authors:** Jiahe Gan, Shi-Ming Li, Shanshan Wu, Kai Cao, Dandan Ma, Xi He, Ziyu Hua, Meng-Tian Kang, Shifei Wei, Weiling Bai, Ningli Wang

**Affiliations:** ^1^Beijing Tongren Eye Center, Beijing Institute of Ophthalmology, Beijing Tongren Hospital, Capital Medical University, Beijing, China; ^2^Beijing Ophthalmology and Visual Science Key Laboratory, Beijing Tongren Eye Center, Beijing Tongren Hospital, Capital Medical University, Beijing, China; ^3^Department of Epidemiology and Health Statistics, Peking University School of Public Health, Beijing, China

**Keywords:** atropine, myopia, efficacy & safety, dose, follow-up

## Abstract

**Purpose:** To evaluate the efficacy and safety of atropine for slowing myopia progression and to investigate whether the treatment effect remains constant with continuing treatment.

**Method:** Studies were retrieved from MEDLINE, EMBASE, and the Cochrane Library from their inception to May 2021, and the language was limited to English. Randomized controlled trials (RCTs) and cohort studies involving atropine in at least one intervention and placebo/non-atropine treatment in another as the control were included and subgroup analysis based on low dose (0.01%), moderate dose (0.01%–<0.5%), and high dose (0.5–1.0%) were conducted. The Cochrane Collaboration and Newcastle-Ottawa Scale were used to evaluate the quality of RCTs and cohort studies, respectively.

**Results:** Twelve RCTs and fifteen cohort studies involving 5,069 children aged 5 to 15 years were included. The weighted mean differences in myopia progression between the atropine and control groups were 0.73 diopters (D), 0.67 D, and 0.35 D per year for high-dose, moderate-dose, and low-dose atropine, respectively (χ^2^ = 13.76; *P* = 0.001, *I*^2^ = 85.5%). After removing studies that provided extreme findings, atropine demonstrated a significant dose-dependent effect on both refractive change and axial elongation, with higher dosages of atropine resulting in less myopia progression (*r* = 0.85; *P* = 0.004) and less axial elongation (*r* = −0.94; *P* = 0.005). Low-dose atropine showed less myopia progression (−0.23 D; *P* = 0.005) and less axial elongation (0.09 mm, *P* < 0.001) in the second year than in the first year, whereas in high-dose atropine more axial elongation (−0.15 mm, *P* = 0.003) was observed. The higher dose of atropine was associated with a higher incidence of adverse effects, such as photophobia with an odds ratio (OR) of 163.57, compared with an OR of 6.04 for low-dose atropine and 8.63 for moderate-dose atropine (*P* = 0.03).

**Conclusion:** Both the efficacy and adverse effects of atropine are dose-dependent in slowing myopia progression in children. The efficacy of high-dose atropine was reduced after the first year of treatment, whereas low-dose atropine had better efficacy in a longer follow-up period.

## Introduction

Myopia has emerged as a serious public health issue with a rapidly increasing prevalence worldwide (1, 2), especially in some Asian areas (3–6). The myopia prevalence reached 52.7% in 2020 among Chinese adolescents, which prompted Chinese governments to implement nationwide myopia control policies including increasing the engagement of children in outdoor activities. However, the deadly outbreak of the coronavirus disease 2019 (COVID-19) pandemic largely reduced opportunities for children to spend time outdoors (7). Prolonged home confinement has brought excessive time for near work and insufficient time outdoors, both of which have been recognized as major environmental risk factors for myopia development (8–10).

Therefore, solutions for myopia management are of great social concern. In recent years, the treatment with different doses of topical atropine has been recognized as currently one of the most effective treatments for myopia (11), and has been applied to more than 60% of children with myopia in Taiwan (12). However, it is still pending approval by the FDA and has remained an off-label treatment in mainland China and most of the western countries since high doses (0.5–1%) of atropine have inevitable ocular side effects, such as cycloplegia, photophobia, allergic reaction, blurred near vision, and accelerated progression on cessation (rebound effect) (13, 14). Therefore, moderate doses (0.01–0.5%) and low doses of atropine (0.01%) have been widely applied in clinical treatment for children with myopia in recent years.

In our previous meta-analysis, we found a difference in efficacy of atropine among different ethnicities, with greater effects in Asians than in white children (15). Then, we conducted the first randomized clinical trials (RCTs) on low-dose atropine in mainland China and found a 34.2% reduction in myopia progression within 1 year (16). However, there are still some uncertainties and controversies. Some studies reported that the efficacy of atropine was dose-related (17), whereas others found that efficacy of atropine was dose-independent within the range of 0.01–1% (13, 18). Most RCTs and cohort studies reported a first-year protective effect on myopia, whereas the Atropine for the Treatment of Myopia 2 (ATOM 2) study showed a better effect of 0.01% atropine treatment in the second year than in the first year, and it is recommended that the initial treatment of 0.01% atropine should last at least 2 years (19). But the evidence is still lacking on whether continuing eyedrops for a longer duration of treatment can produce a continued effect (20, 21). In addition, some eye-care professionals have been concerned that potential side effects (e.g., photophobia) of atropine may affect children's quality of life and reduce compliance, which may influence the efficacy of myopia control. Therefore, an optimal dose of atropine with substantial efficacy and acceptable side effects has remained undetermined. Comparison of different doses is essential to enable clinicians and parents to choose the safest and most effective treatment for myopia control.

In this meta-analysis, we aimed to evaluate the overall efficacy and safety of different doses of atropine with more updated RCTs and cohort studies and to explore the dose-response relationship of atropine. We also investigated whether there was an efficacy difference across different treatment periods.

## Methods

This meta-analysis was performed in compliance with the Preferred Reporting Items for Systematic Reviews and Meta-Analyses (PRISMA) guidelines (eTable 1 in the Supplementary Material) (22).

### Eligibility Criteria

We included comparative studies (i.e., RCTs, and cohort studies) according to the following criteria: (1) a human study investigating the relationship between topical atropine and myopia in school-aged children (between 6 and 15 years); (2) using atropine in at least one intervention and placebo or non-atropine treatment in another as the control; and (3) reporting at least one outcome of interest, including the annual rate of myopia progression and any adverse effects. In addition, the dose of atropine was classified into 3 subgroups: low dose (0.01%), moderate dose (>0.01% to <0.5%), and high dose (0.5–1.0%) (23).

### Search Methods

Data were obtained from MEDLINE, EMBASE, and the Cochrane Library from their inception to May 2021 with language striction in English. We selected RCTs and cohort studies involving atropine in at least one treatment arm and placebo or non-atropine treatment in another as the control that reported myopia progression and/or side effects of atropine therapy for analysis. Medical Subject Headings (MeSH) and the following as keywords: myopia, refractive errors, muscarinic antagonists, cholinergic antagonists, mydriatics, atropine, clinical trial, and humans, as well as some relevant free terms were used for search. Boolean operators “AND,” “OR,” “NOT” were used to combine all search sets. Detailed search strategies are provided in eTable 2 in the Supplementary Material. We also screened clinicaltrials.gov and the reference lists of published reviews to identify additional relevant studies. Exclusion criteria were (at least one of the following): overlapping population; non-human studies; lack of data for outcomes of interest; and studies published as abstracts, reviews, case reports, comments, letters to the editor, and animal research.

### Data Extraction and Quality Assessment

Two investigators (GJH and MDD) independently screened the titles, abstracts, and full-text articles for inclusion using standardized data extraction forms. When the same population was involved in multiple reports, only the latest report was included to avoid duplicated data. Both investigators extracted the study characteristics from each trial: (1) first author, (2) year of publication, (3) study design, (4) country or area, (5) intervention and control, (6) follow-up duration, (7) sample size, (8) baseline characteristics (sex, age, refraction, axial length, dropouts from total number), (9) endpoints (mean change in refraction and axial length), and (10) number of side effects. All disagreements were reviewed by a third investigator (HX). For any missing data, we contacted the authors of the trial reports or used GetData GraphDigitizer 2.24 (http://getdata-graph-digitizer.com) to read data from figures. The list of exclusion studies and reasons for exclusion were shown in eTable 3 in the Supplementary Material. The quality of the selected trials was assessed by the following six aspects following the recommendations of Cochrane collaboration (24) for RCTs: allocation sequence generation, allocation concealment, masking of patients and clinicians, masking of outcome assessors, incomplete outcome data, and selective outcome reporting. Newcastle-Ottawa Quality Assessment Scale items (25) with a “star system” were applied to assess the quality of cohort studies and included 8 items within 3 domains: selection (representativeness), comparability (because of design or analysis), and outcomes (assessment and follow-up). A study can be awarded a maximum of 1 star for each numbered item within the selection and outcome categories and a maximum of 2 stars can be given for comparability, and the total scores range from 0 to 9 stars. Stars of 0–3, 4–6, 7–9 were considered as low, moderate, and high quality, respectively (26).

### Outcome

The efficacy outcome were mean annual changes in refraction [diopters (D)/year], axial length (mm/year), and the number of children showing myopia progression. The safety outcomes were the number of adverse events including photophobia, blurred near vision, and allergy. We also extracted data on photopic and mesopic pupil diameter (mm) and change in accommodation (amplitude/year).

### Statistical Analysis

Data analyses were conducted using Review Manager (Version 5.4; The Cochrane Collaboration, 2020). We calculated the weighted mean difference (MD) and 95% confidence intervals (95% CIs) for different doses of atropine in refractive changes and axial elongation vs. the control group, as well as the odds ratios (ORs) for adverse effects between the atropine and control groups. The effect sizes (ESs) were calculated using the Cohen d formula. ORs with 95% CIs of proportions with fast (>1.0 diopters (D) per year)/slow (<0.5 D/year) myopia progression was also calculated. Heterogeneity was assessed using Cochran's *Q*-test and *I*^2^ statistics. If the heterogeneity was not significant (*p* > 0.1, *I*^2^ < 50.0%), a fixed-effects model was used; otherwise, a random-effects model was used.

Sensitivity analysis was performed by excluding studies with significantly different characteristics to assess their influence on the overall estimates. Subgroup analyses were pre-planned to compare the treatment effects among children with different doses of atropine [low dose (0.01%), moderate dose (>0.01 to <0.5%), high dose (0.5–1.0%)], treatments in control groups (placebo or non-placebo), and ethnicity. Meta-regression analysis was also conducted to identify potential sources of heterogeneity. *P* for interaction was performed using linear mixed effects model, where we built a product term of doses of atropine × ethnicity, as well as a product term of doses of atropine × study design (RCT or cohort study). Publication bias was assessed by visual inspection of funnel plot if the number of retrieved studies was >10. *P* < 0.05 was considered statistically significant for all analyses.

## Results

**Figure 1:**
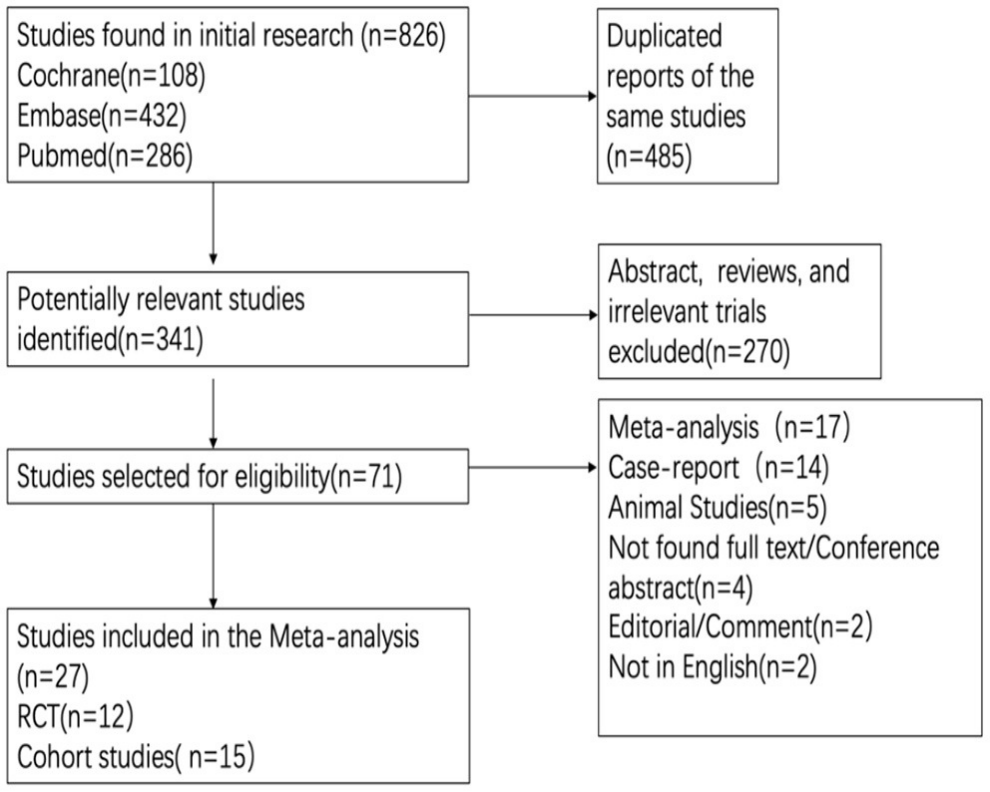
Flowchart of the literature search and study selection.

The search yielded a total of 826 articles, of which 12 RCTs (16, 27–36) and 15 cohort studies (37–51) were included for final analysis (Figure 1). Table 1 details the relevant features of the 27 studies. Briefly, the total sample size of participants included in our study was 5,069, among which 3,024 were received atropine treatment and 2,045 participants were received placebo or non-atropine treatment, with a follow-up period from 12 to 144 months. Concerning geographical location of the studies, 7 studies were conducted in mainland China, 8 in Taiwan, 4 in the United States, 2 in Singapore, 2 in Hong Kong, 2 in Europe, 1 in Japan, and 1 in India, resulting in most participants being Asian. All RCTs were conducted in Asia, among which Wei et al. (16) provided the first placebo-controlled RCT data for low-dose atropine in mainland China.

**Table 1:** Characteristics of the studies included in the meta-analysis.

**References**	**Country/Area**	**Study design**	**Follow-up (months)**	**Age (years)**	**Intervention**		**Baseline refraction, diopter mean (SD)**
					**Experimental group (atropine dose, %)**	**Control group**	
Yen et al. (35)	Taiwan, China	RCT	12	6–14	1.00 every other night	Placebo	−1.52 ± 0.92
Shih et al. (30)	Taiwan, China	RCT	24	6–13	0.5% + bifocals	0.5% tropicamide nightly + full correction	−4.41 ± 1.47
					0.25% + partially undercorrected glasses		
					0.1% + full eyeglass correction		
Shih et al. (31)	Taiwan, China	RCT	18	6–13	0.5% + multifocal lenses	Multifocal lenses	−3.26 ± 0.15
Chua et al. (27)	Singapore	RCT	24	7–12	1.00	Placebo	−3.36 ± 1.38
Chia et al. (28)	Singapore	RCT	48	6–12	0.5, 0.1, 0.01	–	0.38 ± 0.60
Yi et al. (36)	China	RCT	12	6–12	1.00	Placebo	−1.23 ± 0.32
Wang et al. (32)	China	RCT	12	5–10	0.50	Placebo	−1.30 ± 0.40
Yam et al. (34)	China	RCT	12	4–12	0.05, 0.025, 0.01	Placebo	−1.00 or less
Wei et al. (16)	China	RCT	12	6–12	0.01	Placebo	−2.52 ± 1.33
Zhu et al. (33)	China	RCT	48	6–12	1	Placebo	−3.82 ± 0.44
Saxena et al. (52)	India	RCT	12	6–14	0.01	Placebo	−3.5 ± 1.3
Hieda et al. (53)	Japan	RCT	24	6–12	0.01	Placebo	−1.00 to −6.00
Bedrossian (37)	USA	Cohort	33	8–12	1	Blank	−0.50 or less
Chou et al. (38)	Taiwan, China	Cohort	38	7–14	0.5	Self-contrast	−6.25 to −12.00
Kennedy et al. (45)	USA	Cohort	144	6–15	1	Blank	−1.49
Lee et al. (40)	Taiwan, China	Cohort	20	6–12	0.05	Blank	−1.58 ± 1.37
Fan et al. (39)	Hongkong, China	Cohort	12	5–10	1	Blank	−5.18 ± 2.05
Fang et al. (43)	Taiwan, China	Cohort	18	6–12	0.025	Blank	−0.31 ± 0.45
Wu et al. (50)	Taiwan, China	Cohort	54	6–12	0.05	Blank	−2.45 ± 1.63
Lin et al. (41)	China	Cohort	12	8–15	1.00	Self-contrast	−1.92 ± 0.91
Clark and Clark (42)	USA	Cohort	13	6–15	0.01	Blank	−2.00 ± 1.60
Lee et al. (46)	Taiwan, China	Cohort	12	5–14	0.125, 0.25	Blank	−1.45 ± 0.69
Polling et al. (54)	Europe	Cohort	12	8–13	0.50	Withdraw population	−6.70 ± 3.60
Moon and Shin (44)	Korea	Cohort	12	5–14	0.01, 0.025, 0.05	Self-contrast	−3.84 ± 2.47
Larkin et al. (47)	USA	Cohort	24	6–15	0.01	Blank	−3.10 ± 1.90
Sacchi et al. (49)	Europe	Cohort	12	5–14	0.01	Blank	−3.00 ± 2.23
Fu et al. (51)	China	Cohort	12	6–12	0.01, 0.02	Blank	−2.76 ± 1.47

### Risk of Bias Assessment

Bias for the included RCTs is presented in eTable 4 in the Supplementary Material. There were two RCTs (30, 35) assessed as high risk of bias due to unclear randomization, inadequate loss to follow-up and without blinding. The quality of the included cohort studies was generally high according to the Newcastle-Ottawa Scale items (26) (eTable 5 in the Supplementary Material).

### Effect of Atropine on the Annual Rate of Myopia Progression

Changes in refraction from 12 RCTs and 15 cohort studies were obtained. Since no difference between RCTs and cohort studies was observed in low-dose, moderate-dose, and high-dose subgroups (eFigure 1 in the Supplementary Material; all *P* > 0.05 in the test for subgroup difference), we thus evaluated the effects of atropine by combining RCTs and cohort studies to provide larger samples for different doses.

**Figure 2:**
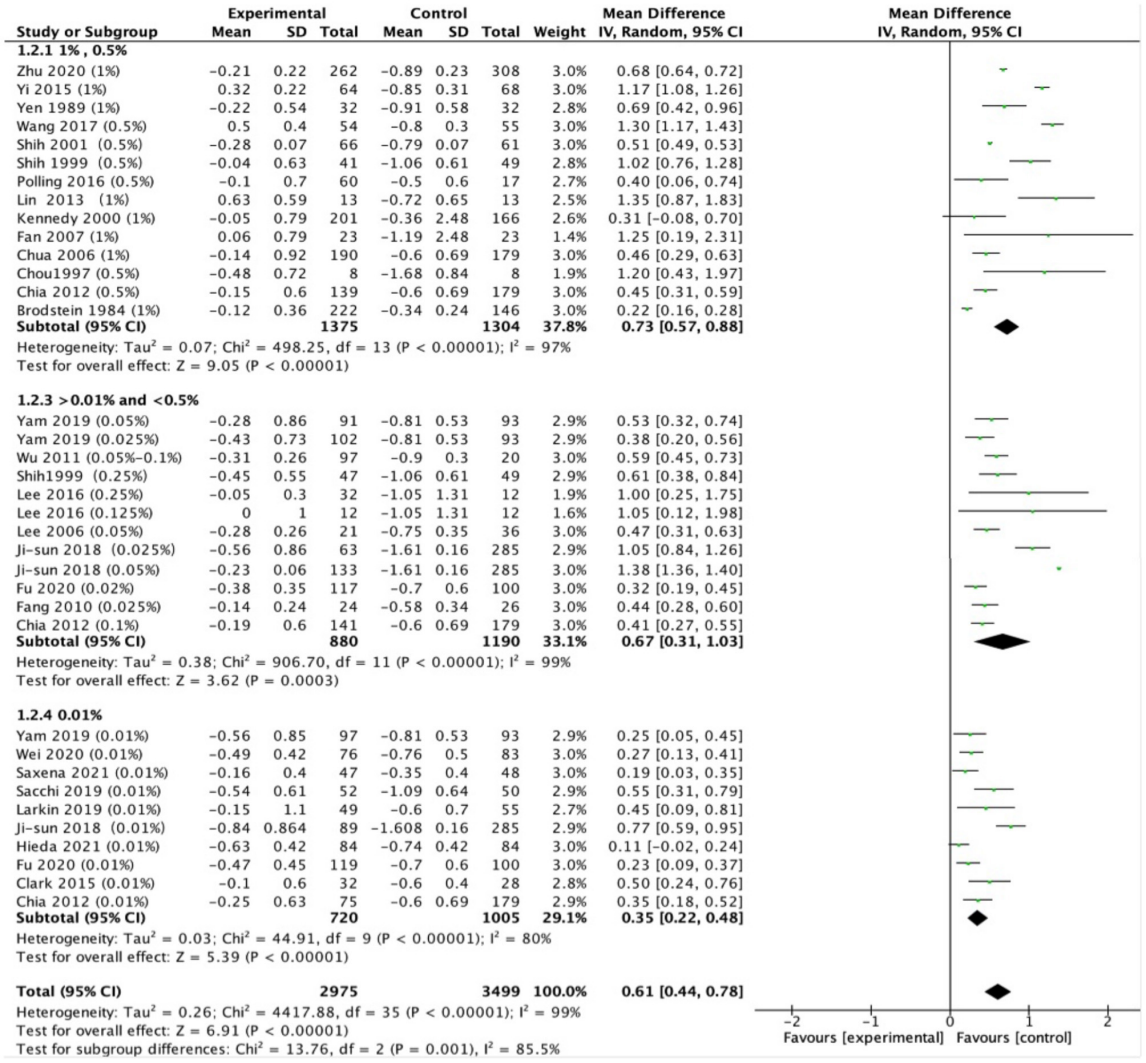
Effects of different doses of atropine on slowing myopia progression (diopters/year).

**Figure 3:**
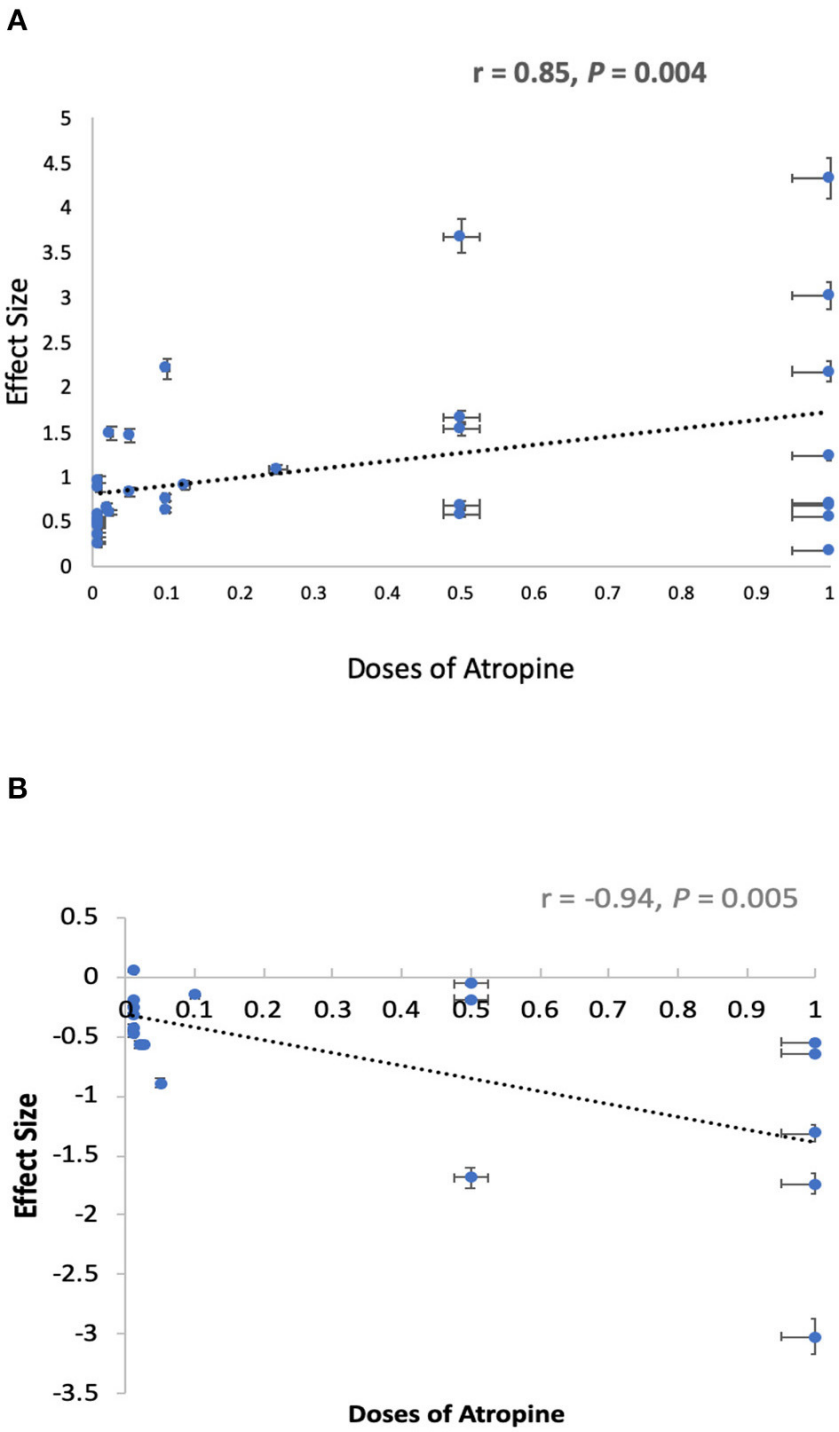
Graphical summary of effect sizes of different doses of atropine for prevention of myopia progression. **(A)** Effect sizes of different doses of atropine for prevention of refraction change. **(B)** Effect sizes of different doses of atropine for prevention of axial elongation.

The pooled data revealed significantly less progression in refraction for low-dose (MD, 0.35D per year; 95% CI, 0.22–0.48D per year; *P* < 0.001), moderate-dose (MD, 0.67D per year; 95% CI, 0.31–1.03D per year; *P* < 0.001), and high-dose (MD, 0.73D per year; 95% CI, 0.57–0.98D per year; *P* < 0.001) atropine groups than control groups (Figure 2). There was a statistically significant difference in refraction changes among various doses of atropine within this range (χ^2^ = 13.76; *P* = 0.001 for subgroup difference; *I*^2^ = 85.5%). The effect sizes showed a large treatment effect in different dose atropine groups (Figure 3). We observed no correlation between a dose and treatment effect (*r* = 0.665; *P* = 0.051). However, when the study by Moon and Shin (44) was excluded because of extreme findings due to the dose of atropine was prescribed according to the myopia progression rate of the patients, the treatment effect of mean annual refraction change was significantly correlated with the dose of atropine (*r* = 0.85; *P* = 0.004).

Heterogeneity of the meta-analysis was significant (*P* < 0.001, *I*^2^ = 99%; Figure 2). We did subgroup analysis based on different treatments in control groups (placebo or non-placebo) and still observed significant heterogeneity in low-dose, moderate-dose, and high-dose subgroups (eFigure 2 in the Supplementary Material). In addition, a significant difference was found between Asian and white individuals in high dose atropine studies (*P* < 0.001), suggesting ethnicity might be a source of additional heterogeneity (eFigure 3 in the Supplementary Material). And this was supported by our finding that there was a significant interactive effect between doses of atropine and ethnicity on mean annual refraction change (Table 2; *P*-interaction = 0.006). Further analysis found that there was significant difference in refraction changes among various doses of atropine in Asian population (*P* = 0.008).

**Table 2:** Test for interaction on mean annual refraction change by doses of atropine, ethnicity, and study design.

**Characteristics**	**No. of studies**	**MD (95% CI)**	***P*-interaction**
**Doses of atropine**			
High	14	0.73 (0.57, 0.88)	
Moderate	12	0.67 (0.31, 1.03)	
Low	10	0.35 (0.22, 0.48)	
**Ethnicity**			
Asian patients	21	0.65 (0.46, 0.83)	**0.006** ^*^
White patients	6	0.39 (0.23, 0.54)	
**Study design**			
RCT	18	0.55 (0.43, 0.67)	0.4508^†^
Cohort studies	19	0.68 (0.36, 1.01)	

*MD, mean difference. Bold type indicates statistically significant*.

* *Test for interaction between doses of atropine and ethnicity on mean annual refraction change*.

† *Test for interaction between doses of atropine and study design on mean annual refraction change*.

### Effects on Changes in Axial Length

**Figure 4:**
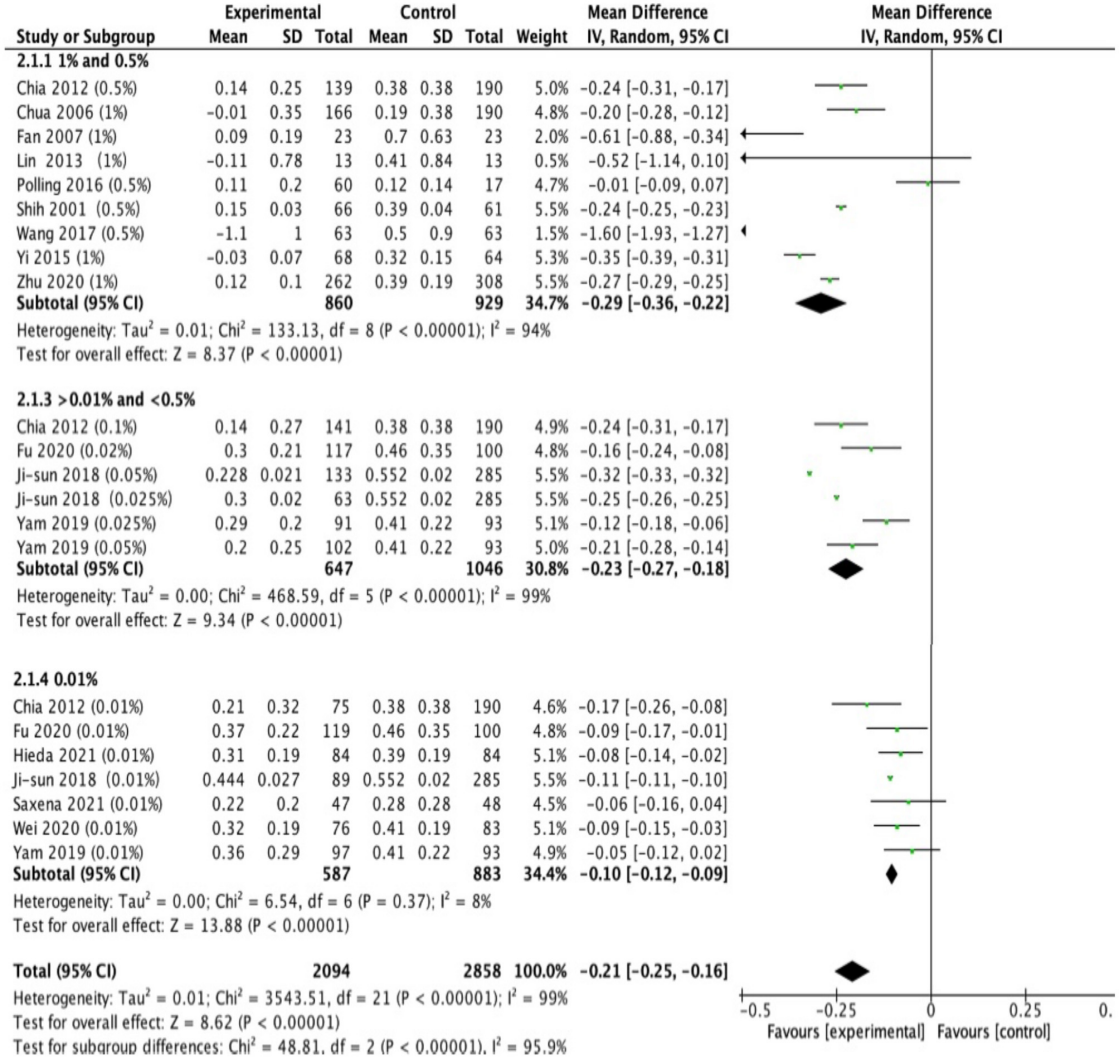
Effects of different doses of atropine on slowing axial elongation (mm/year).

Thirteen studies reported changes in axial length. The analyses showed that the MD was −0.29 mm in high-dose atropine studies (95% CI, −0.36 to −0.22 mm; *P* < 0.001), −0.23 mm in moderate-dose atropine studies (95% CI, −0.27 to −0.18 mm; *P* < 0.001) and −0.10 mm in low-dose atropine studies (95% CI, −0.12 to −0.09 mm; *P* < 0.001; Figure 4). A statistically significant difference in axial elongation across various doses of atropine within this range (χ^2^ = 48.81; *P* < 0.001 for subgroup difference; *I*^2^ = 95.9%) with significant (*P* < 0.001) heterogeneity (*I*^2^ = 99%). The effect sizes showed a large treatment effect for annual axial length change in different dose atropine groups (Figure 3). When the study by Moon and Shin (44) was excluded because of extreme findings, a significant dose and treatment effect on annual axial elongation was observed (*r* = −0.94; *P* = 0.005; Figure 3).

### Rapid Myopia Progression (>1.0 D per Year)

Six RCTs and seven cohort studies reported the number of children with rapid myopia progression (>1.0 D per year). The odds ratio (OR) of rapid myopia progression was significantly lower in atropine compared to control in both RCTs (OR, 0.13; 95% CI, 0.10–0.18; *P* < 0.001) and cohort studies (OR, 0.19; 95% CI, 0.10–0.3; *P* < 0.001). The RCTs and cohort studies were combined in subsequent analyses because no difference was found between them (χ^2^ = 0.93; *P* = 0.33 for subgroup difference; *I*^2^ = 0%). High-dose atropine showed the lowest OR for rapid myopia progression (95% CI, 0.08–0.13; *P* < 0.001), followed by 0.16 in moderate-dose atropine (95% CI, 0.08–0.31; P < 0.001), and 0.29 in low-dose atropine (95% CI, 0.18–0.47; *P* < 0.001) (eFigure 4A in the Supplementary Material with significant difference among three groups (χ^2^ = 14.88; *P* < 0.001 for subgroup difference; *I*^2^ =86.6%).

### Slow Myopia Progression (<0.5 D per Year)

The number of children with slow myopia progression was assessed in 6 RCTs and 7 cohort studies (<0.5 D per year). All of the different concentrations of atropine had a higher OR of slow myopic progression relative to control in both RCTs (OR, 6.84; 95% CI, 4.15–11.29; *P* < 0.001) and cohort studies (OR, 6.05; 95% CI, 3.09–11.84; *P* < 0.001). The combined analyses showed that the OR for atropine slowing myopia progression was 6.98 in high-dose (95% CI, 0.08–0.13 mm; *P* < 0.001), 7.67 in moderate-dose (95% CI, 3.67–16.00; *P* < 0.001), and 3.50 in low-dose (95% CI, 2.02–6.06; *P* < 0.001; eFigure 4B in the Supplementary Material).

### Treatment Efficacy With Different Treatment Durations

**Figure 5:**
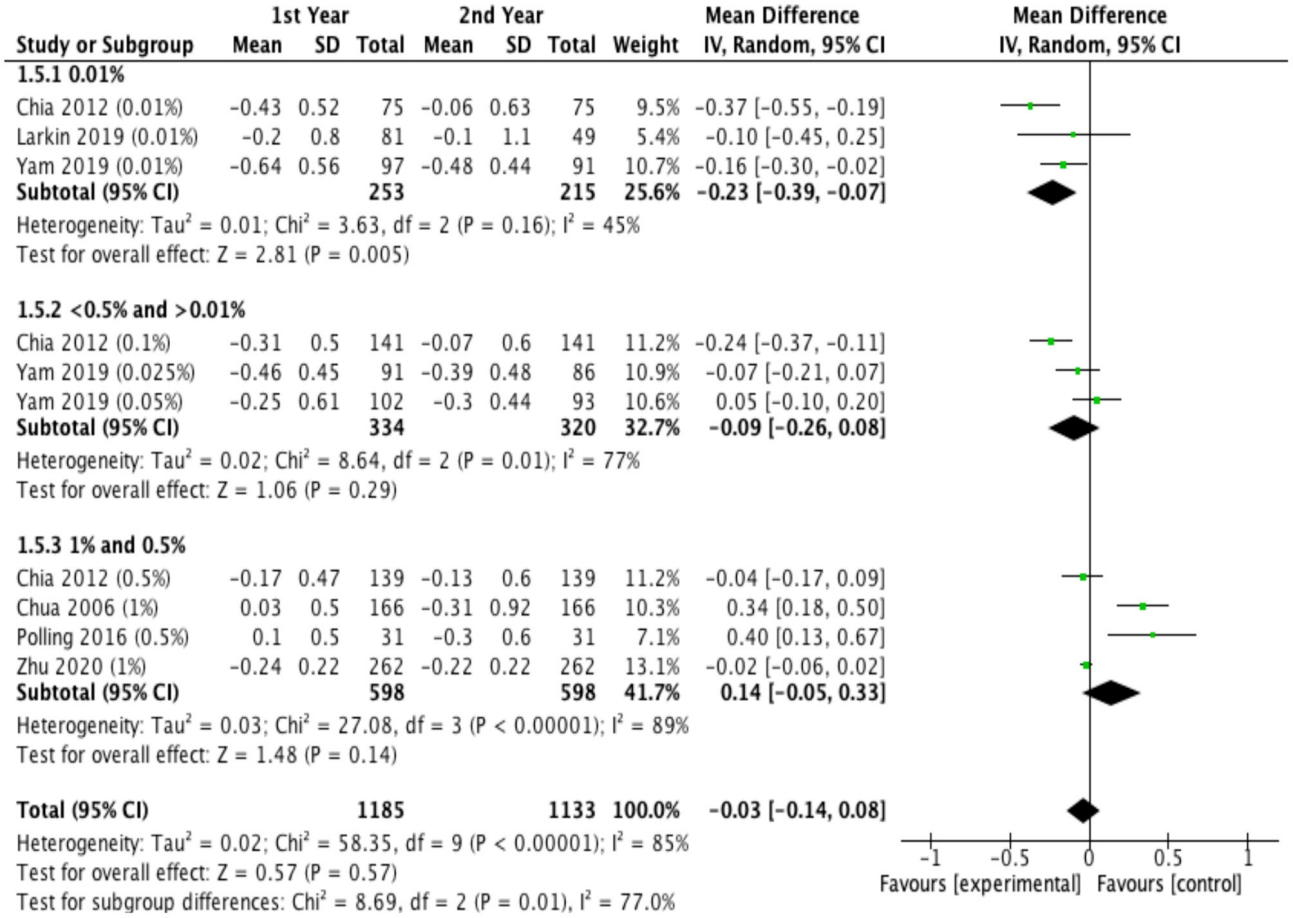
Effects of different doses of atropine on refraction changes in the first and second years of treatment (diopters/year).

**Figure 6:**
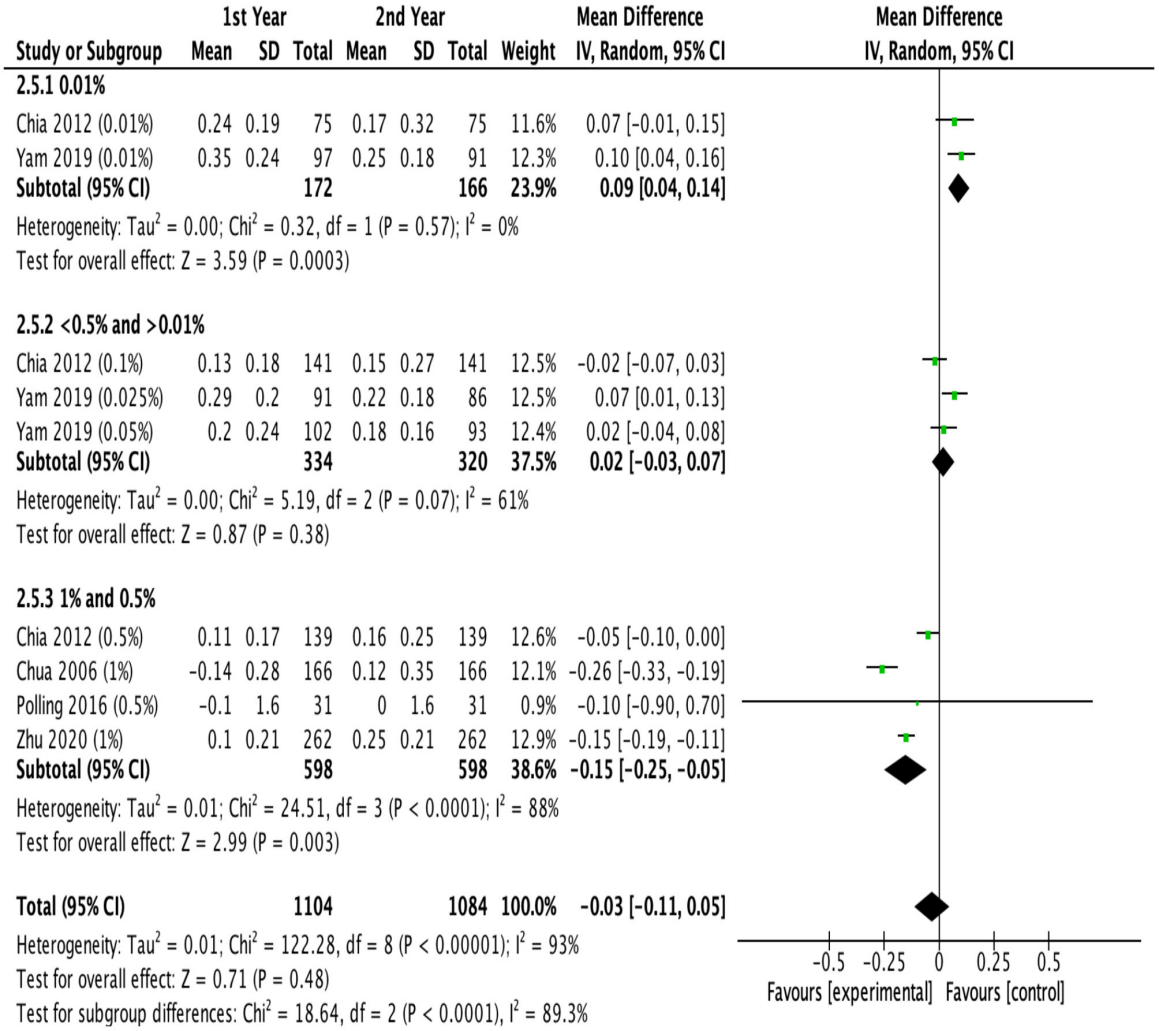
Effects of different doses of atropine on axial elongation in the first and second years of treatment (mm/year).

Figure 5 showed the difference in efficacy of atropine between the second year and the first year. Children treated with low-dose atropine appeared to benefit more in the second year than in the first year (refraction change: −0.23 D, 95% CI, −0.39 to −0.07, *P* = 0.005; axial elongation: 0.09 mm, 95% CI, 0.04–0.14, *P* = 0.003). However, high-dose atropine showed less efficacy in the second year with a greater progression of refraction (refraction change: 0.14 D, 95% CI, −0.05–0.33, *P* = 0.14) and significantly more axial elongation (axial length change: −0.15 mm, 95% CI, −0.25 to −0.05, *P* = 0.003) than in the first year of treatment (Figure 6).

### Side Effects

A total of 17 studies reported the incidence of side effects. Table 3 showed the most frequently reported side effects of topical atropine, including photophobia [388 of 1,757 (25.1%)], blurred near vision [144 of 1,633 (7.5%)], and allergic reaction [49 of 1,387 (2.9%)].

**Table 3:** Adverse events in the atropine groups vs control group during the treatment of myopia in children.

**Outcomes**	**No. of studies**	**No. of patients**	**Odds Ratio** **(95% CI)**	***I^**2**^***
Photophobia	5 RCTs	388/1,757 vs. 15/2,325	16.69 (5.37–51.9)	70.7%
	9 Cohort			
Blurred near vision	4 RCTs	144/1,633 vs. 0	17.16 (7.97, 36.95)	0
	6 Cohort			
Allergy	5 RCTs	49/1,387 vs. 21/1,483	2.24 (1.37–3.64)	77.0%
	2 Cohort			

#### Photophobia

We found that all of the different concentrations of atropine had a higher OR of photophobia relative to the control (OR = 16.69, 95% CI = 5.37 to 51.9, eFigure 7A in the Supplementary Material). Specifically, high-dose atropine showed the highest OR for photophobia (OR = 163.57, 95% CI = 19.5–1,372.0), followed by moderate-dose atropine (OR = 8.63, 95% CI = 2.19–33.96), and low-dose atropine (OR = 6.04, 95% CI = 1.39–26.23), showing an increase in the rate of this adverse effect with dose escalation (χ^2^ = 6.83; *P* = 0.03 for subgroup difference, eFigure 5A in the Supplementary Material). The incidence of photophobia was statistically significant correlated with the dose of atropine (*r* = 0.86; *P* = 0.001).

#### Blurred Near Vision

The OR for poor near visual acuity with low-, moderate- and high-dose atropine was 17.45 (95% CI = 4.04–75.44), 20.52 (95% CI, 6.12–68.86), and 39.65 (95% CI = 11.39–137.97), respectively (eFigure 5B in the Supplementary Material).

#### Allergy

The OR for allergies with low-, moderate, and high-dose atropine was 1.27 (95% CI = 0.47–3.39), 1.28 (95% CI = 0.63–2.59), and 10.86 (95% CI = 2.95–40.04), respectively (eFigure 5C in the Supplementary Material), revealing an increase in the rate of this adverse effect with dose escalation (χ^2^ = 8.68; *P* = 0.01 for subgroup difference).

### Effects on Accommodation and Pupil Size

We summarized the effects of atropine on accommodation amplitude in eFigure 6 in the Supplementary Material. A significant effect on accommodative amplitudes was found among groups receiving different doses of atropine, revealing a smaller decline in accommodation amplitude with low-dose atropine than with higher-dose atropine (−1.80 D for low-dose, −2.7 D for moderate-dose, and −5.75 D for high-dose atropine; *P* < 0.001).

As exemplified in eFigure 7 in the Supplementary Material, there was no significant difference in pupillary enlargement under photopic conditions with low-dose atropine compared with moderate-dose atropine (*P* = 0.91). Meanwhile, the number of studies examining changes in pupil size under mesopic conditions was too small (only 1 study in each subgroup) to evaluate the effect of atropine on pupil enlargement.

### Evaluation of the Sensitivity, Regression Analysis, and Publication Bias

We conducted sensitivity analyses on MD in refraction change, excluding studies (1) published before 2000, (2) with baseline mean refraction <-4D or (3) with a high risk of bias (eFigure 8 in the Supplementary Material). We noted that the conclusions on the outcome did not change substantially after omitting studies with significantly different characteristics. The potential sources of heterogeneity were further explored through meta-regression analysis (eTable 6 in the Supplementary Material). While meta-regression analysis found ethnicity as the only statistically significant moderator with greater effects on slowing mypia progression in Asian than in white children (0.37, 95% CI 0.04–0.70).

A funnel plot for publication bias test for the outcome showed an asymmetric left-right distribution, indicating the possibility of publication bias. Factors such as insufficient sample sizes and the lack of reporting on negative results were the possible causes of publication biases (eFigure 9 in the Supplementary Material).

## Discussion

In this meta-analysis, we compared the results from 12 RCTs and 15 cohort studies and confirmed that there was significantly less myopia progression (MD = 0.70 D) and slower axial elongation (MD = −0.21 mm) in the atropine group than in the control group. After excluding the study by Ji-sun et al. (43), we found that the effectiveness of atropine was related to its dose, and this was consistent with previous meta-analyses conducted in 2011 and 2020 (17, 55).

Moreover, different doses of atropine had a significantly lower OR in children with rapid myopia progression (OR = 0.16, 95% CI = 0.11–0.23, eFigure 4A in the Supplementary Material) and a significantly higher OR in children with slow myopia progression (OR = 5.88, 95% CI = 3.86–8.95, eFigure 4B in the Supplementary Material), which was consistent with Ha et al. (18) and our previous meta-analysis published in 2014 (15).

Previous studies have demonstrated that most myopia interventions, including multifocal lenses, orthokeratology, and atropine, lost their effectiveness after the first year of treatment (21, 23, 56). However, the review that concluded that the treatment efficacy of atropine diminished over time relied on only a single prospective study of low-dose atropine and moderate-dose atropine, and therefore, the conclusion was preliminary (56). Previous studies generally presented the treatment efficacy of atropine at different time points as a cumulative effect relative to baseline. Here, we broke down the treatment efficacy into individual time segments to better illustrate the annual myopia progression during the first year and the second year of treatment. Our study suggested for the first time that the effects of low-dose atropine showed better efficacy in slowing myopia progression during the second year of treatment in protecting both refraction and axial elongation, which was consistent with the conclusion of ATOM2 study (19); moderate-dose atropine showed no difference in efficacy in the second year compared with the first year, and high-dose atropine showed less efficacy during the second year. In addition, ATOM2 study reported that compared with high-dose atropine, low-dose atropine showed the smallest rebound effect after ceasing the treatment and ended with the lowest myopic progression over the entire 3-year period (19). Therefore, low-dose atropine showed a sustained effect on inhibiting the progression of myopia in the long-term treatment. Since axial elongation naturally slows with time, it is reasonable to believe that the efficacy of high-dose atropine wanes over time. However, it is difficult to know whether the observed reductions in axial elongation with low-dose atropine during the second year were simply a function of this deceleration in growth or a change in the efficacy of atropine (21, 57). The treatment efficacy of atropine should be further investigated with longer follow-up. However, the control groups in many studies of atropine on myopia control generally given a specific dose for 1–2 years and then switched to other doses for ethical reasons, which makes long-term follow-up more difficult.

Previously, few data were available for the quantitative assessment of adverse effects of topical atropine, except the meta-analysis conducted by Gong et al. (13) and Ha et al. (18), which showed that a higher dose of atropine led to an increasing number of adverse effects. Our results also demonstrated that the side effects of atropine, such as photophobia was dose-dependent. Due to the small number of reported literature on some other side effects, such as systematic symptoms (58), decline of cognitive function (59), meta-analysis cannot be done yet. Among these, systematic symptoms and decline of cognitive function have only been found in oral atropine drugs, whereas topical atropine eyedrops could hardly enter the systematic circulation by pressing the inner canthus while applying the eyedrops. And a few clinical trials have shown that children who used atropine eyedrops with 1 or 2 year follow-up periods did not show dry eye symptoms (27, 36, 48), elevated intraocular pressure (27), retinal photic injury (60, 61), though animal research found that 1% atropine eyedrops 4 times a day could induce dry eye in rabbits (62).

There have been several meta-analyses investigating various doses of atropine treatment in myopia control. A previous meta-analysis by Song et al. (55), Li et al. (15) and Gong et al. (13) included only 6, 11, and 19 studies, respectively. Recently, a network meta-analysis conducted by Ha et al. built up hierarchies of atropine treatment in terms of efficacy and safety among the 8 concentrations (18). But the analysis included only 16 RCTs, without comparing the treatment difference during the first year and second year.

There are some limitations in the present study. First, although this meta-analysis had established strict inclusion and exclusion criteria, the heterogeneity was still high after using the subgroup analysis. Because of insufficient data on some concentrations, different doses of atropine were combined in high dose and moderate dose studies in this meta-analysis, which might be a source of heterogeneity. And RCTs and cohort studies were combined to investigate the overall effects of different doses, although cohort studies showed similar effects to RCTs. Heterogeneity also result from ethnicity, since meta-regression analysis found that atropine had greater effects on slowing mypia progression in Asian than in white children. We then conducted sensitivity analysis by omitting studies with significantly different characteristics (the year of publication year, baseline refraction, and quality of studies) and found that the outcomes remained stable. However, the publication bias analysis results showed that there might exist publication bias, so the results should be interpreted with caution. Second, more than half of the included studies did not report adverse reactions; thus, the reports on adverse effects in the included studies were not comprehensive. Third, the efficacy of atropine in our study was reported during the treatment period, and the follow-up periods significantly varied among the trials. Fourth, most of the studies evaluated were conducted among Asians. Differences between Asian and Caucasian individuals in their response to interventions for myopia progression were significant (eFigure 3, eTable 6 in the Supplementary Material). Fifth, some of the results were based on data from limited studies. For example, the effects of different doses of atropine on refraction changes in the first and second years of treatment, there were only 2 studies in some subgroups, so the results should be interpreted with caution.

Despite the limitations mentioned above, the strength of this study includes a comprehensive quantitative analysis of both efficacy and safety on varying doses of atropine. This will provide a valuable reference for the clinical application of atropine since large clinical trials for comparison of all atropine doses are unlikely to be carried out. The ideal dose of atropine in myopia control should balance efficacy and safety with the best risk/benefit ratios. In this study, low dose atropine (0.01%) demonstrated valid efficacy in retarding refraction changes and axial elongation relative to the control group with minimal side effects and showed better efficacy in a longer follow-up period. Thus, 0.01% atropine should be advocated in the treatment for slowing myopia progression.

## Conclusions

This meta-analysis suggests that both the efficacy and the adverse effects of atropine eyedrops are dose-dependent and that the efficacy of high-dose atropine on slowing myopia progression was reduced after the first year of treatment, whereas low-dose atropine may have better efficacy in a longer follow-up period.

## Data Availability Statement

The original contributions presented in the study are included in the article/Supplementary Material, further inquiries can be directed to the corresponding author/s.

## Author Contributions

JG, S-ML, and NW were responsible for conceptualizing, designing, data collection, extraction, interpretation, manuscript drafting, statistical analysis, and conducting the study. JG and SWu were responsible for data collection, extraction, and critical revisions of the manuscript. KC helped on the manuscript revision. JG, DM, XH, ZH, M-TK, SWe, and WB were responsible for data interpretation, manuscript drafting, supervision, and critical revisions of the manuscript for important intellectual content. S-ML and NW are the guarantors of this article and takes full responsibility for this study. All authors contributed to the article and approved the submitted version.

## Funding

This works was supported by grants from the Capital Health Research and Development of Special (2020-2-1081), Beijing Natural Science Foundation (JQ20029), Beijing Talents Found (2016000021223ZK28), and the National Natural Science Foundation of China (82071000).

## Author Disclaimer

This article's contents are solely the responsibility of the authors.

## Conflict of Interest

The authors declare that the research was conducted in the absence of any commercial or financial relationships that could be construed as a potential conflict of interest.

## Publisher's Note

All claims expressed in this article are solely those of the authors and do not necessarily represent those of their affiliated organizations, or those of the publisher, the editors and the reviewers. Any product that may be evaluated in this article, or claim that may be made by its manufacturer, is not guaranteed or endorsed by the publisher.
