# Supplementary material for: Varying Dose of Atropine in Slowing Myopia Progression in Children Over Different Follow-Up Periods by Meta-Analysis
Source: Front Med (Lausanne). 2022 Jan 13;8:756398. doi: 10.3389/fmed.2021.756398 (PMC8792607; doi:10.3389/fmed.2021.756398)
Supplement: Supplementary file 1 [file Table_1.docx]

***Supplementary Material***

**eTable 1. PRISMA Checklist**

**eTable 2. Search Strategy**

**eTable 3. Exclusion studies list and exclusion reason**

**eTable 4. Quality assessment of RCTs included in the meta-analysis According to the Cochrane Collaboration Tool**

**eTable 5. Quality assessment of cohort studies included in the meta-analysis using Newcastle-Ottawa Quality Assessment Scale**

**eTable 6. Meta-regression Analysis of Mean Difference in Refraction Change and Axial Length Change**

**eFigure 1.** **Forest Graph of the Effect of Atropine** **on Mean Annual Refraction Change From Both RCTs and Cohort Studies**

**eFigure 2. Forest** **Graph of the Effect of Atropine on Mean Annual Refraction Change compared with Placebo and Non-placebo Treatment**

**eFigure 3.** **Forest** **Graph of the Effect of High Doses of Atropine on Mean Annual Refraction Change in Asian and White Patients**

**eFigure 4. Forest Graph of the effect of Atropine on Proportions of Children with Myopia Progression**

**eFigure 5. Forest Graph and Incidence of Adverse Events**

**eFigure 6. Forest Graph of the Effect of Different Doses of Atropine on Accommodation Amplitude**

**eFigure 7. Forest Graph of the Effect of Different Doses of Atropine on Photopic**

**Pupil Size**

**eFigure 8. Sensitivity Analysis**

**eFigure 9. Funnel Plots for Publication Bias**

**eTable 1. PRISMA Checklist**

| **Section and Topic** | **Item #** | **Checklist item** |  |
| --- | --- | --- | --- |
| **TITLE** | | |  |
| Title | 1 | Identify the report as a systematic review. | **1** |
| **ABSTRACT** | | |  |
| Abstract | 2 | See the PRISMA 2020 for Abstracts checklist. | 3 |
| **INTRODUCTION** | | |  |
| Rationale | 3 | Describe the rationale for the review in the context of existing knowledge. | 5-6 |
| Objectives | 4 | Provide an explicit statement of the objective(s) or question(s) the review addresses. | 3;6 |
| **METHODS** | | |  |
| Eligibility criteria | 5 | Specify the inclusion and exclusion criteria for the review and how studies were grouped for the syntheses. | 6-7 |
| Information sources | 6 | Specify all databases, registers, websites, organisations, reference lists and other sources searched or consulted to identify studies. Specify the date when each source was last searched or consulted. | 7 |
| Search strategy | 7 | Present the full search strategies for all databases, registers and websites, including any filters and limits used. | eTable2 |
| Selection process | 8 | Specify the methods used to decide whether a study met the inclusion criteria of the review, including how many reviewers screened each record and each report retrieved, whether they worked independently, and if applicable, details of automation tools used in the process. | 7-8 |
| Data collection process | 9 | Specify the methods used to collect data from reports, including how many reviewers collected data from each report, whether they worked independently, any processes for obtaining or confirming data from study investigators, and if applicable, details of automation tools used in the process. | 7 |
| Data items | 10a | List and define all outcomes for which data were sought. Specify whether all results that were compatible with each outcome domain in each study were sought (e.g. for all measures, time points, analyses), and if not, the methods used to decide which results to collect. | 8 |
|  | 10b | List and define all other variables for which data were sought (e.g. participant and intervention characteristics, funding sources). Describe any assumptions made about any missing or unclear information. | Table 1 |
| Study risk of bias assessment | 11 | Specify the methods used to assess risk of bias in the included studies, including details of the tool(s) used, how many reviewers assessed each study and whether they worked independently, and if applicable, details of automation tools used in the process. | 7; 9 |
| Effect measures | 12 | Specify for each outcome the effect measure(s) (e.g. risk ratio, mean difference) used in the synthesis or presentation of results. | 8 |
| Synthesis methods | 13a | Describe the processes used to decide which studies were eligible for each synthesis (e.g. tabulating the study intervention characteristics and comparing against the planned groups for each synthesis (item #5)). | 8 |
|  | 13b | Describe any methods required to prepare the data for presentation or synthesis, such as handling of missing summary statistics, or data conversions. | 8 |
|  | 13c | Describe any methods used to tabulate or visually display results of individual studies and syntheses. | Table 1 |
|  | 13d | Describe any methods used to synthesize results and provide a rationale for the choice(s). If meta-analysis was performed, describe the model(s), method(s) to identify the presence and extent of statistical heterogeneity, and software package(s) used. | 9 |
|  | 13e | Describe any methods used to explore possible causes of heterogeneity among study results (e.g. subgroup analysis, meta-regression). | 9 |
|  | 13f | Describe any sensitivity analyses conducted to assess robustness of the synthesized results. | 9 |
| Reporting bias assessment | 14 | Describe any methods used to assess risk of bias due to missing results in a synthesis (arising from reporting biases). | 9 |
| Certainty assessment | 15 | Describe any methods used to assess certainty (or confidence) in the body of evidence for an outcome. | 8-9 |
| **RESULTS** | | |  |
| Study selection | 16a | Describe the results of the search and selection process, from the number of records identified in the search to the number of studies included in the review, ideally using a flow diagram. | 12-13; Figure 1 |
|  | 16b | Cite studies that might appear to meet the inclusion criteria, but which were excluded, and explain why they were excluded. | eTable 3 |
| Study characteristics | 17 | Cite each included study and present its characteristics. | Table1 |
| Risk of bias in studies | 18 | Present assessments of risk of bias for each included study. | eTable 4  eTable 5 |
| Results of individual studies | 19 | For all outcomes, present, for each study: (a) summary statistics for each group (where appropriate) and (b) an effect estimate and its precision (e.g. confidence/credible interval), ideally using structured tables or plots. | Figure 2-5 |
| Results of syntheses | 20a | For each synthesis, briefly summarise the characteristics and risk of bias among contributing studies. | 10 |
|  | 20b | Present results of all statistical syntheses conducted. If meta-analysis was done, present for each the summary estimate and its precision (e.g. confidence/credible interval) and measures of statistical heterogeneity. If comparing groups, describe the direction of the effect. | 10-13 |
|  | 20c | Present results of all investigations of possible causes of heterogeneity among study results. | 10; 16 |
|  | 20d | Present results of all sensitivity analyses conducted to assess the robustness of the synthesized results. | 13-14 |
| Reporting biases | 21 | Present assessments of risk of bias due to missing results (arising from reporting biases) for each synthesis assessed. | 13 |
| Certainty of evidence | 22 | Present assessments of certainty (or confidence) in the body of evidence for each outcome assessed. | 13-14 |
| **DISCUSSION** | | |  |
| Discussion | 23a | Provide a general interpretation of the results in the context of other evidence. | 14-16 |
|  | 23b | Discuss any limitations of the evidence included in the review. | 16 |
|  | 23c | Discuss any limitations of the review processes used. | 16 |
|  | 23d | Discuss implications of the results for practice, policy, and future research. | 17 |
| **OTHER INFORMATION** | | |  |
| Registration and protocol | 24a | Provide registration information for the review, including register name and registration number, or state that the review was not registered. | NA |
|  | 24b | Indicate where the review protocol can be accessed, or state that a protocol was not prepared. | NA |
|  | 24c | Describe and explain any amendments to information provided at registration or in the protocol. | NA |
| Support | 25 | Describe sources of financial or non-financial support for the review, and the role of the funders or sponsors in the review. | 2 |
| Competing interests | 26 | Declare any competing interests of review authors. | 2 |
| Availability of data, code and other materials | 27 | Report which of the following are publicly available and where they can be found: template data collection forms; data extracted from included studies; data used for all analyses; analytic code; any other materials used in the review. | ­17-22 |

NA=Not Available.

*From:*  Page MJ, McKenzie JE, Bossuyt PM, Boutron I, Hoffmann TC, Mulrow CD, et al. The PRISMA 2020 statement: an updated guideline for reporting systematic reviews. BMJ 2021;372:n71. doi: 10.1136/bmj.n71

**eTable 2. Search Strategy**

## PubMed (from inception to May 2021)

| **Search number** | **Query** | **Results** |
| --- | --- | --- |
| #1 | Myopia[Mesh] | 25,273 |
| #2 | Muscarinic Antagonists[MeSH] | 60,402 |
| #3 | Cholinergic Antagonists[MeSH] | 89,986 |
| #4 | (((((Myopia*[Title/Abstract]) OR (Myopic*[Title/Abstract])) OR (Refractive errors*[Title/Abstract])) OR (Muscarinic Antagonists[Title/Abstract])) OR (Cholinergic Antagonists[Title/Abstract])) OR ( Mydriatics *[Title/Abstract]) | 26,944 |
| #5 | #1 OR #2 OR #3 OR #4 | 635,015 |
| #6 | progress* or slow* or retard* or effect* or treat* or efficacy* | 14,334,615 |
| #7 | (randomized controlled trial [pt] OR controlled clinical trial [pt] OR randomized [tiab] | 834,924 |
| #8 | #6 OR #7 | 16,943,923 |
| #9 | #5 AND 38 | 2,049 |
| #10 | #9 AND ((humans[Filter]) AND (:2021/5/03[pdat])) | 286 |

## EMBASE (from inception to May 2021)

| **Search number** | **Query** | **Results** |
| --- | --- | --- |
| #1 | ‘Myopia’/exp | 26,218 |
| #2 | ‘Myopic’ | 2,478 |
| #3 | ‘shortsighted’/exp | 1,325 |
| #4 | Refraction error’/exp OR ‘Refractive error’ OR ‘Refractive errors’ | 15,495 |
| #5 | #1 OR #2 OR #3 OR #4 | 37,524 |
| #6 | ‘mydriatic agent’/exp OR ‘mydriat*’ OR ‘Muscarinic Receptor Blocking Agent’/exp OR ‘muscarinic antagonist’ OR ‘Cholinergic Receptor Blocking Agent’/exp OR ‘anti cholinergic’ OR ‘Atropine’ | 103,465 |
| #7 | progress* or slow* or retard* or effect* or treat* or efficacy | 16,711,797 |
| #8 | #6 AND #7 | 74,108 |
| #9 | #5 AND #8 | 703 |
| #10 | #9 AND 'human'/de | 600 |
| #11 | #9 AND 'human'/de AND [embase]/lim NOT ([embase]/lim AND [medline]/lim) | 432 |

## Cochrane library (from inception to May 2021)

| **Search number** | **Query** | **Results** |
| --- | --- | --- |
| #1 | MeSH descriptor: [myopia] explode all trees | 2659 |
| #2 | MeSH descriptor: [Refractive Errors] explode all trees | 1703 |
| #3 | MeSH descriptor: [(short OR near*) sight*] explode all trees | 40 |
| #4 | (#1 OR #2 OR #3) | 4406 |
| #5 | (atropine*):ti,ab,kw | 3752 |
| #6 | MeSH descriptor: [Mydriatics] explode all trees | 539 |
| #7 | MeSH descriptor: [Muscarinic Antagonists] explode all trees | 1805 |
| #8 | (cholinergic next antagonist*):ti,ab,kw | 428 |
| #9 | (#5 OR #6 OR #7 OR #8) | 6524 |
| #10 | (progress*):ti,ab,kw OR (slow*):ti,ab,kw OR (retard*):ti,ab,kw OR (efficacy *):ti,ab,kw OR (effect*):ti,ab,kw | 15237 |
| #11 | #4 AND #9 AND #10 | 108 |

**eTable 3. Exclusion studies list and exclusion reason**

|  | Reason for exclusion |
| --- | --- |
| Luu CD(2005)^1^ | No myopic progression rate reported |
| Liang CK(2008)^2^ | No placebo or blank control group: comparing the effect of auricular acupoints and atropine |
| Chia A(2009)^3^ | No myopic progression rate reported: effect of topical atropine on astigmatism |
| Tong L(2009)^4^ | No myopic progression rate reported: effect on myopia progression after cessation of atropine |
| Cooper J(2013)^5^ | No myopic progression rate reported: a transient experiment |
| Chia A(2013)^6^ | No myopic progression rate reported: effect of topical atropine on electroretinogram |
| Kumaran A(2015)^7^ | Duplicated data with Chia 2012. |
| Loughman J(2016)^8^ | No myopic progression rate reported: a transient 5-days experiment |
| Diaz-Llopis M(2018)^9^ | Not in English: only abstract available |
| Kinoshita N(2018)^10^ | No placebo control group: comparing the effect of orthokeratology and atropine |
| Kaymak(2019)^11^ | No myopic progression rate reported |
| Tan Q(2019)^12^ | No placebo control group: comparing the effect of orthokeratology and atropine |
| Lutz(2019) ^13^ | No control group. |

**eTable 4. Quality assessment of RCTs included in the meta-analysis According to the Cochrane Collaboration Tool**

**A.** Risk of bias in individual trials.

**
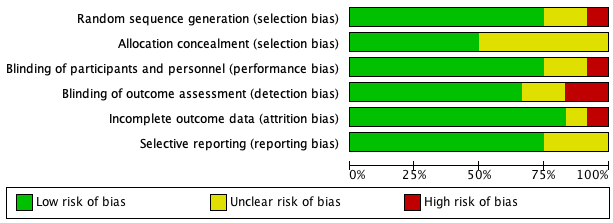
**

**B. Risk of bias across included trials weighted by sample sizes.**

**
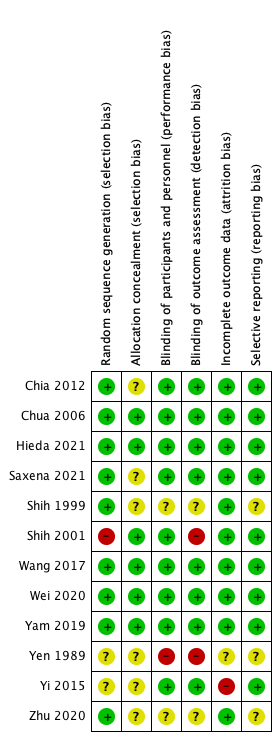
**

**eTable 5. Quality assessment of cohort studies included in the meta-analysis using Newcastle-Ottawa Quality Assessment Scale**

| Study | | Selection | | | | Comparability | Outcome | | |  |  |  |  |  |  |  |  |  |
| --- | --- | --- | --- | --- | --- | --- | --- | --- | --- | --- | --- | --- | --- | --- | --- | --- | --- | --- |
|  | Exposed cohort representative | | Nonexposed cohort selection | Exposure ascertainment | Outcome not present at start |  | Assessment | Follow-up length | Follow-up adequacy |  |  |  |  |  |  |  |  |  |
| Brodstein 1984 | * | | * | * | * | * | * | * | * |  |  |  |  |  |  |  |  |  |
| Chou 1997 | * | | * | * | * | - | * | * | * |  |  |  |  |  |  |  |  |  |
| Kennedy 2000 | * | | * | * | * | ** | * | * | * |  |  |  |  |  |  |  |  |  |
| Lee 2006 | * | | * | * | * | ** | * | * | * |  |  |  |  |  |  |  |  |  |
| Fan 2007 | * | | * | * | * | ** | * | * | * |  |  |  |  |  |  |  |  |  |
| Fang 2010 | * | | * | * | * | ** | * | * | * |  |  |  |  |  |  |  |  |  |
| Wu 2011 | * | | * | * | * | ** | * | * | * |  |  |  |  |  |  |  |  |  |
| Lin 2013 | * | | * | * | * | ** | * | * | * |  |  |  |  |  |  |  |  |  |
| Clark 2015 | * | | * | * | * | * | * | * | * |  |  |  |  |  |  |  |  |  |
| Lee 2016 | * | | * | * | * | * | * | * | * |  |  |  |  |  |  |  |  |  |
| Moon JS 2018 | * | | * | * | * | ** | * | * | * |  |  |  |  |  |  |  |  |  |
| Larkin 2019 | * | | * | * | * | ** | * | * | * |  |  |  |  |  |  |  |  |  |
| Sacchi 2019 | * | | * | * | * | * | * | * | * |  |  |  |  |  |  |  |  |  |
| Fu 2020 | * | | * | * | * | ** | * | * | * |  |  |  |  |  |  |  |  |  |

* indicates score. A study can be awarded a maximum of one star for each numbered item within the Selection and Outcome categories. A maximum of two stars can be given for Comparability.

**eTable 6. Meta-regression Analysis of Mean Difference in Refraction Change and Axial Length Change**

| **Variable** |  | **Pooled estimate (95% CI)** | **p-value** |
| --- | --- | --- | --- |
| **MD in** **refraction change** | Publication year^*^ | 0.0009 (-0.0121 to 0.0139) | 0.89 |
|  | Baseline mean refractive error^†^ | -0.0327(-0.1167 to 0.0514) | 0.78 |
|  | Sample size^‡^ | -0.0001 (-0.0010 to 0.0139) | 0.44 |
|  | Ethnicity^#^ | 0.3680 (0.0359 to 0.7001) | **0.03** |
|  | Risk of bias^§^ | -0.1415 (-0.2942 to 0.0111) | 0.07 |
| **MD in axial length change** | Baseline mean refractive error^†^ | 0.0006 (-0.0003 to 0.0015) | 0.19 |
|  | Sample size^‡^ | -0.0582 (-0.1525 to 0.0362) | 0.22 |
|  | Risk of bias^§^ | -0.1650 (-0.0109 to 0.3408) | 0.06 |

MD = mean difference. Bold type indicates statistically significant

^*^publishing before 2000, ^†^baselinee mean refractive error less than -4 diopters, ^‡^ sample size fewer than 50 participants, ^#^conducted in Asian patients ^§^high risk of bias.

**eFigure 1. Forest Graph of the Effect of Atropine on Slowing Myopia Progression per Year From Both RCTs and Cohort Studies**

1. **Mean Annual Refraction Change in High-Dose studies in RCTs and Cohort Studies**

**
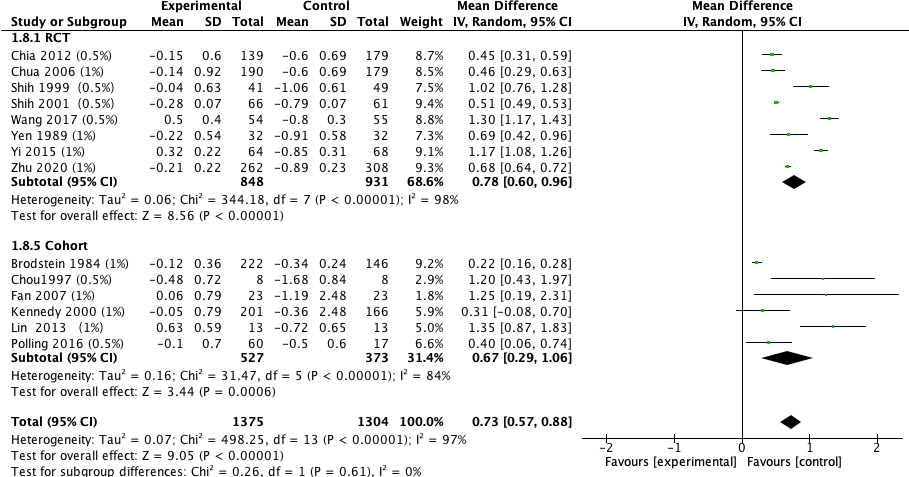
**

1. **Mean Annual Refraction Change in Moderate-Dose studies in RCTs and Cohort Studies**

**
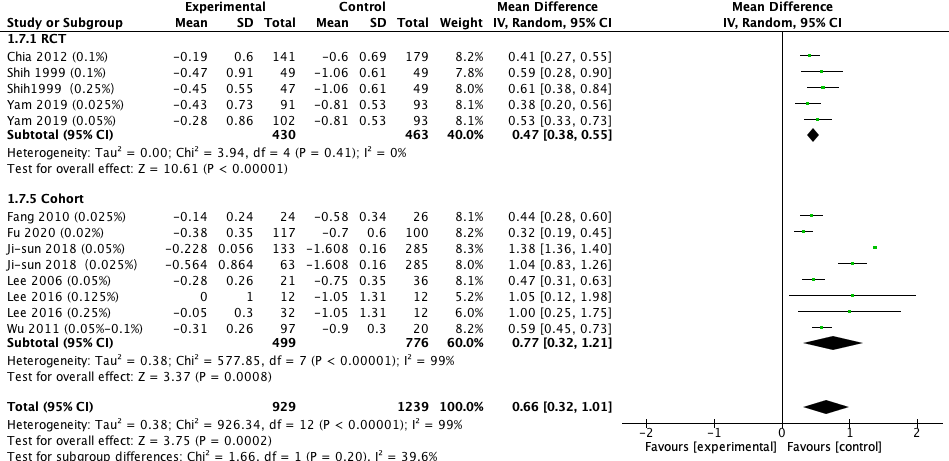
**

1. **Mean Annual Refraction Change in Low-Dose studies in RCTs and Cohort Studies**

**
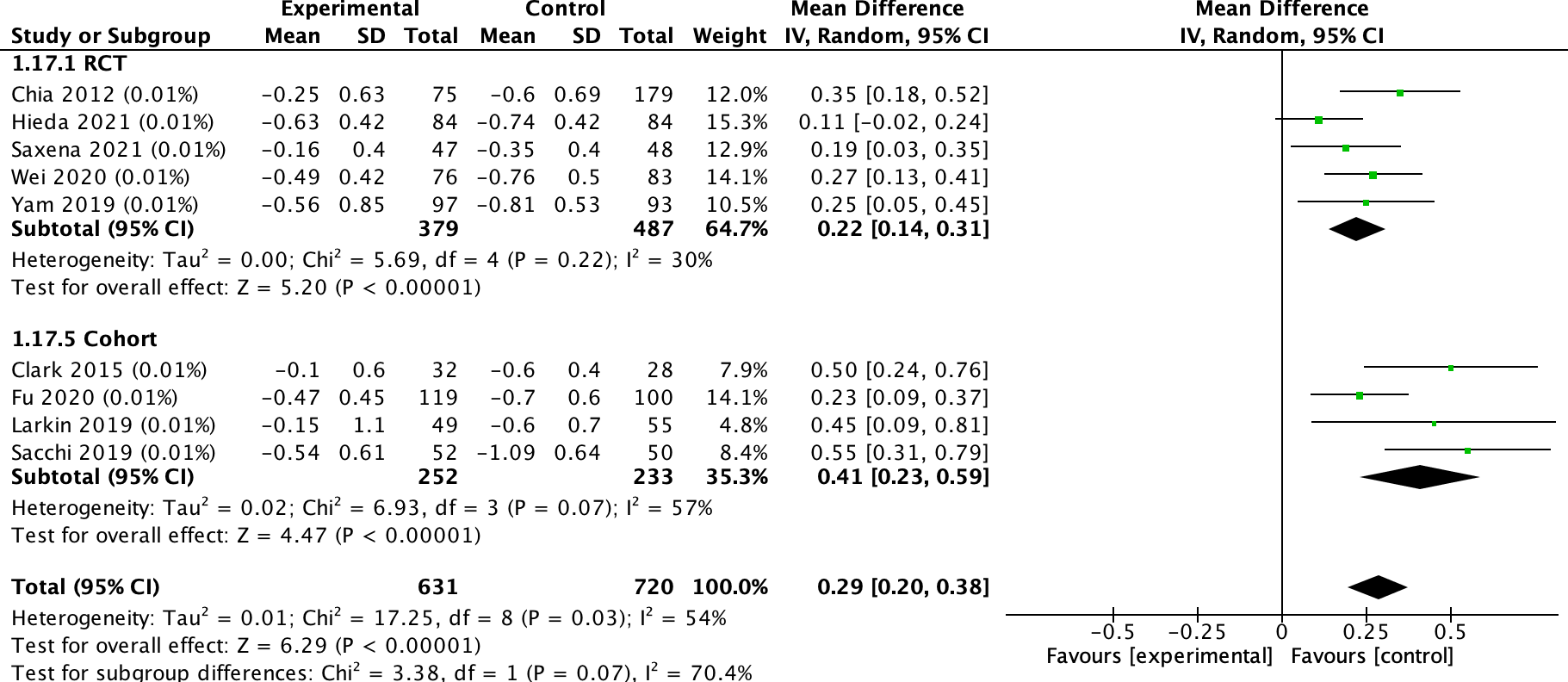
**

**eFigure 2. Forest Graph of the Effect of Atropine on Slowing Myopia Progression per Year Compared with Placebo and Non-placebo Treatment**

**A.** **Mean Annual Refraction Change in High-Dose studies in Placebo and Non-placebo Treatment
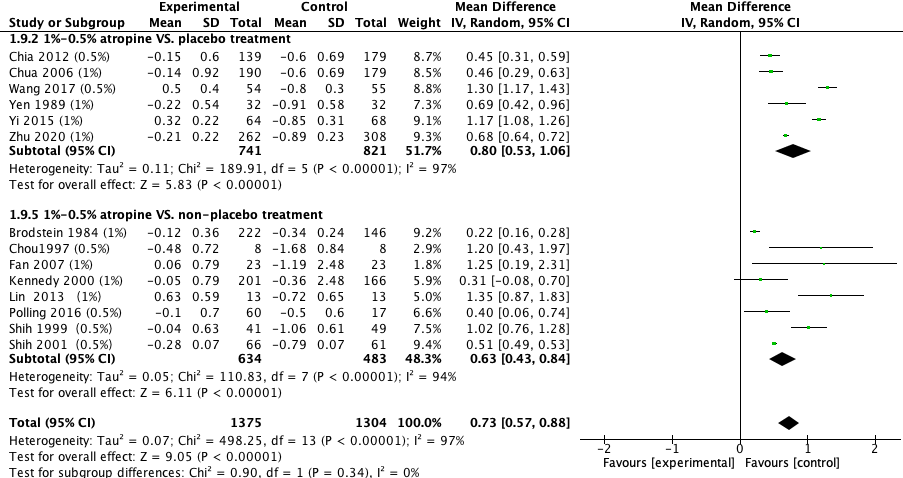
**

**B.** **Mean Annual Refraction Change in Moderate-Dose studies in Placebo and Non-placebo Treatment
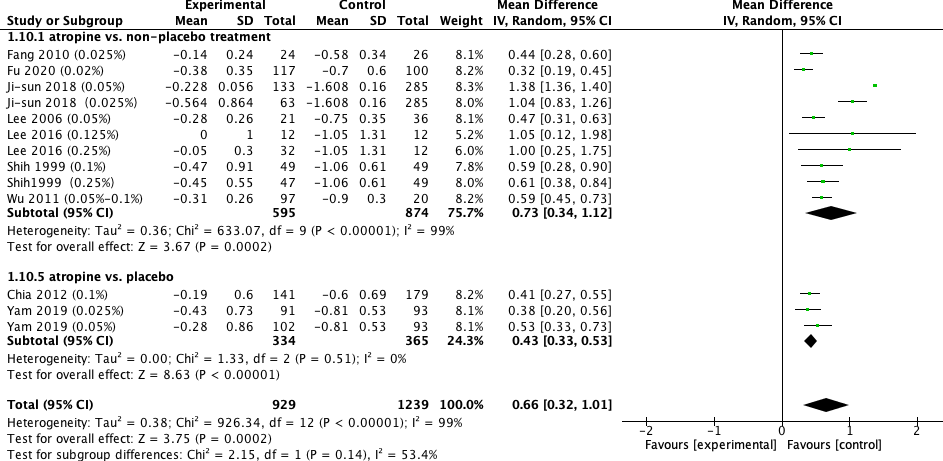
**

**C.** **Mean Annual Refraction Change in Low-Dose studies in Placebo and Non-placebo Treatment**

**
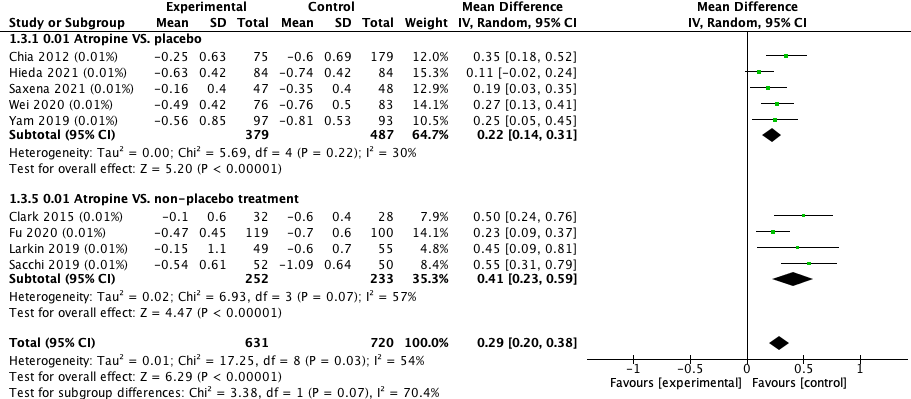
**

**eFigure 3. Forest Graph of the Effect of High Doses of Atropine on Slowing Myopia Progression per Year in Asian and White Patients
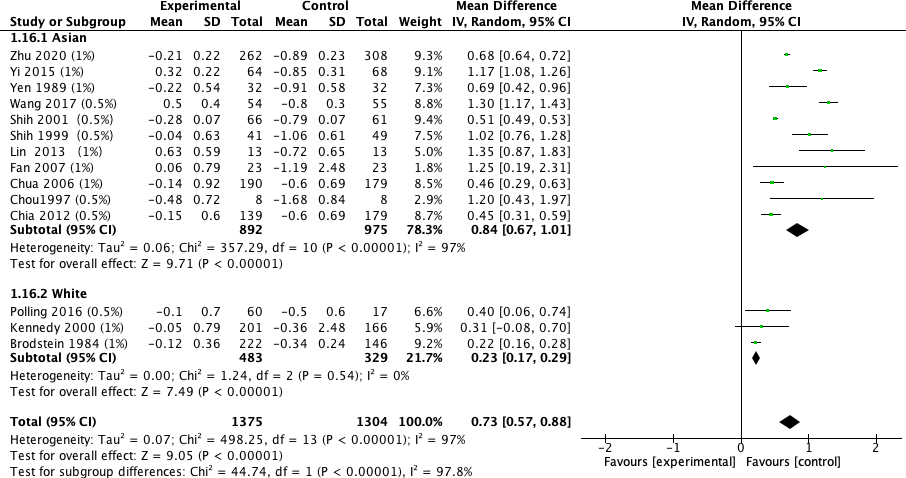
**

**eFigure 4. Forest Graph of the Effect of Atropine on Proportions of Children with Myopia Progression**

1. **Forest Graph of the Effect of Atropine on Proportions of Children with Rapid Myopia Progression (>1.0D/Y).**

**
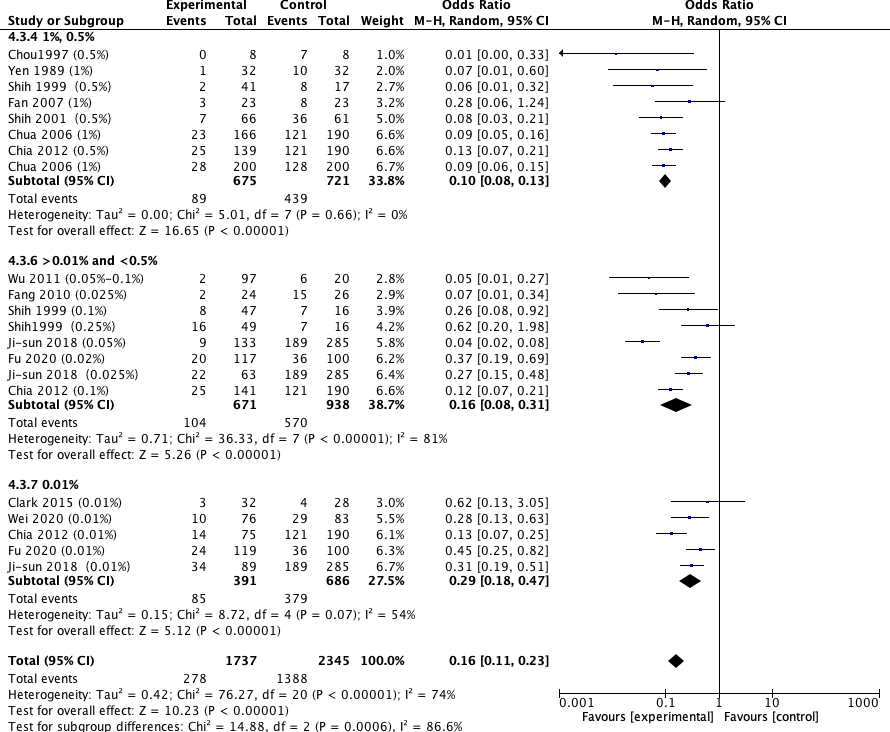
**

**B.** **Forest Graph of the Effect of Atropine on Proportions of Children with Slow Myopia Progression (<0.5D/Y)**

**
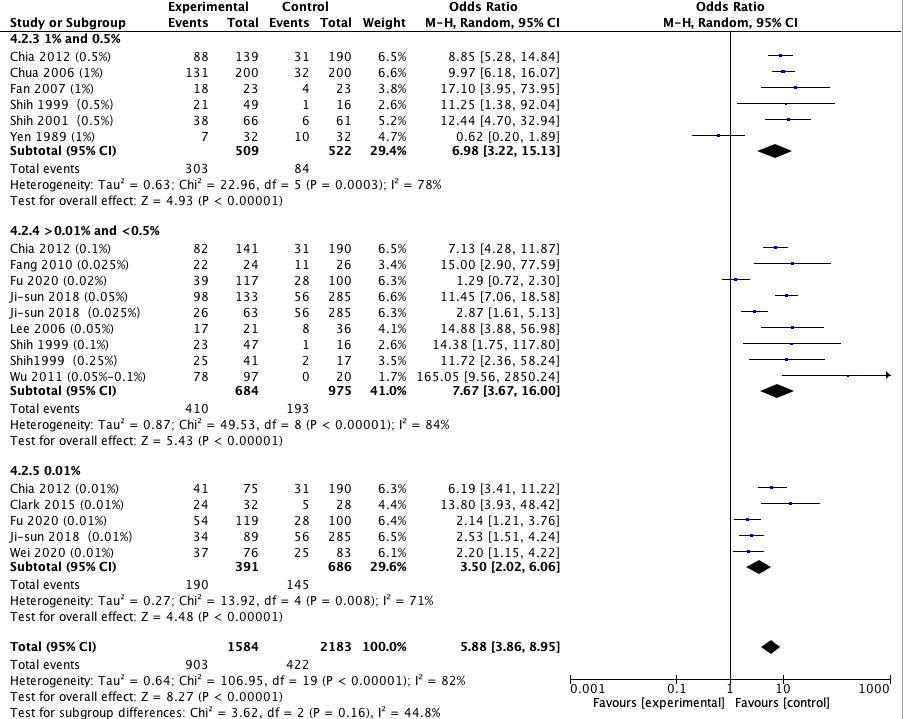
**

**eFigure 5. Forest Graph and Incidence of Adverse Events**

1. **Forest Graph and Incidence of Photophobia**

**
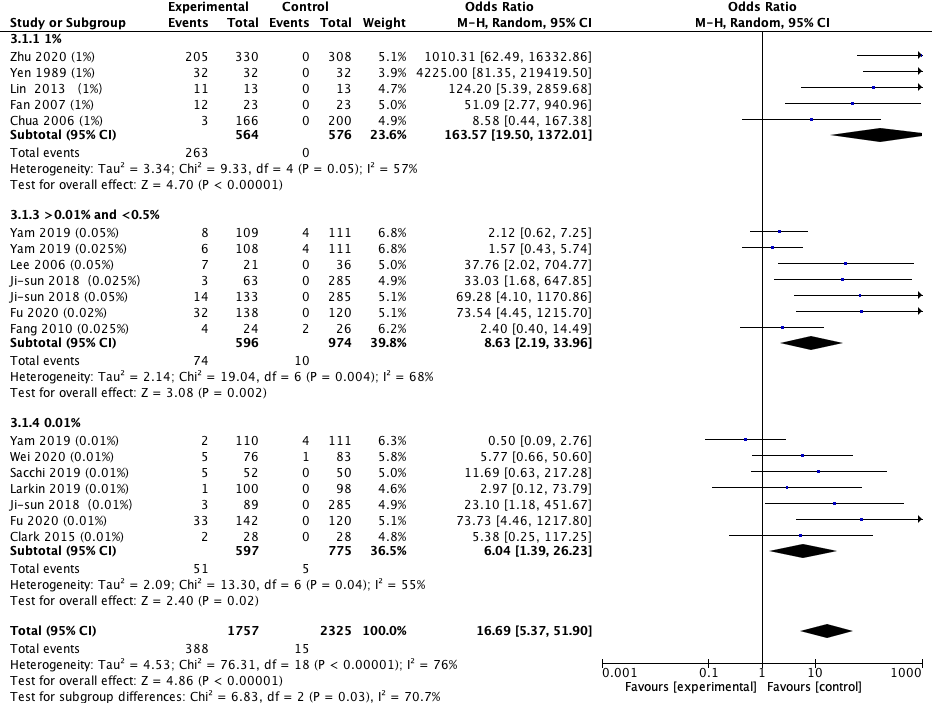
**

**B. Forest Graph and Incidence of Poor Near Visual Acuity**

**
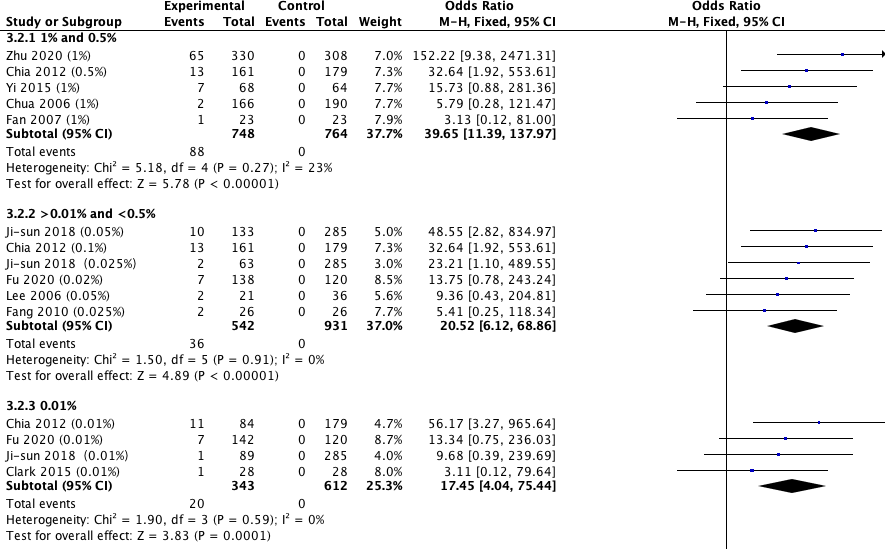
**

**C. Forest Graph and Incidence of Allergy**

**
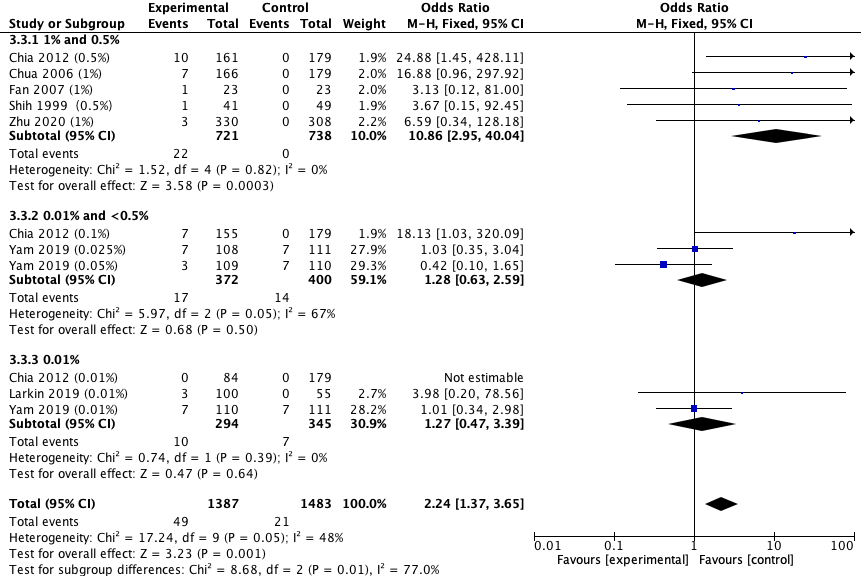
**

**eFigure 6. Forest Plots of the Effect of Different Doses of Atropine on Accommodation Amplitude**

**
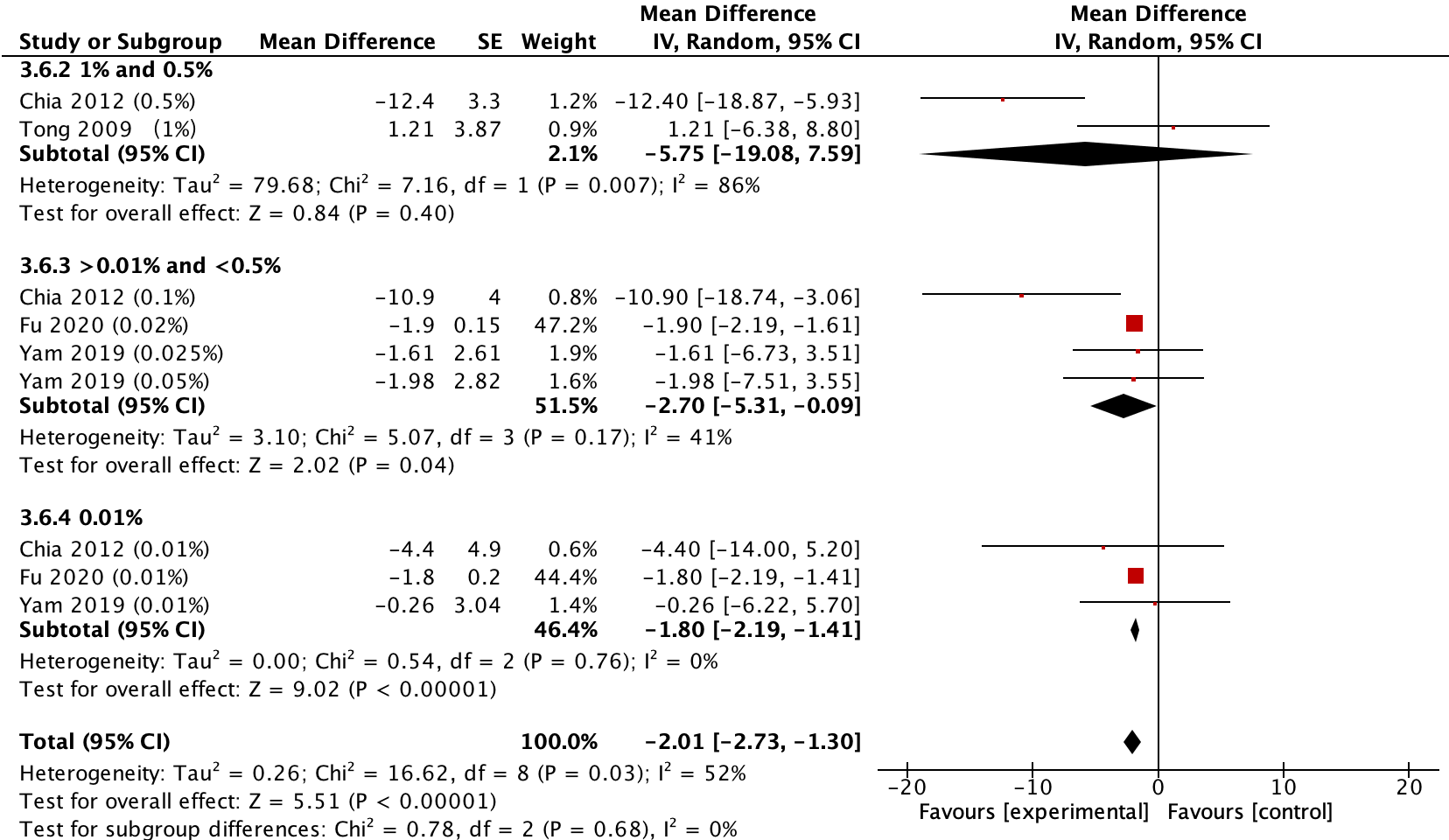
**

**eFigure 7. Forest Plots of the Effect of Different Doses of Atropine on Photopic**

**Pupil Size**

**
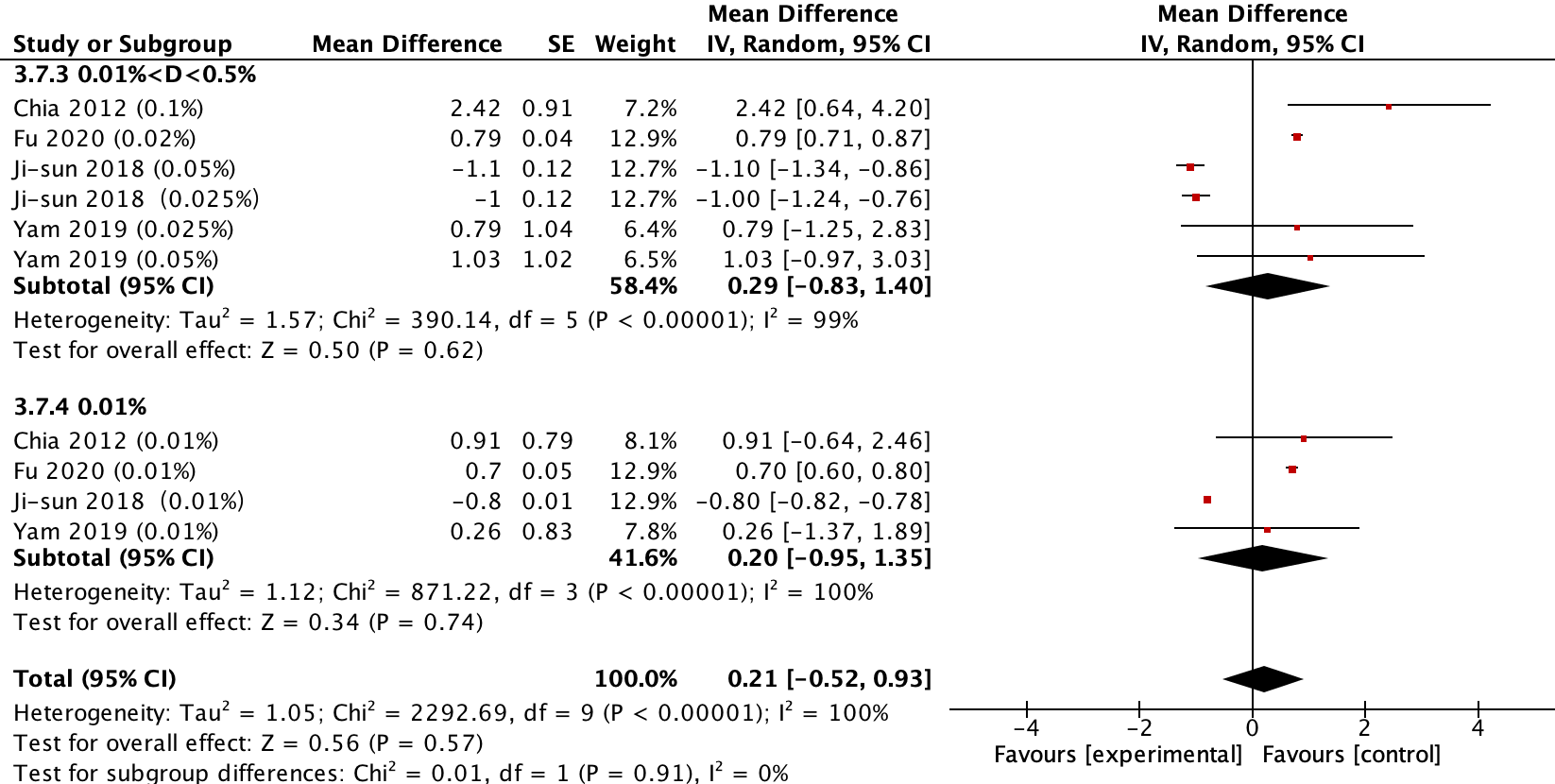
**

**eFigure 8. Sensitivity Analysis**

1. **Sensitivity Analysis after Removing Studies Published Before 2000**

**
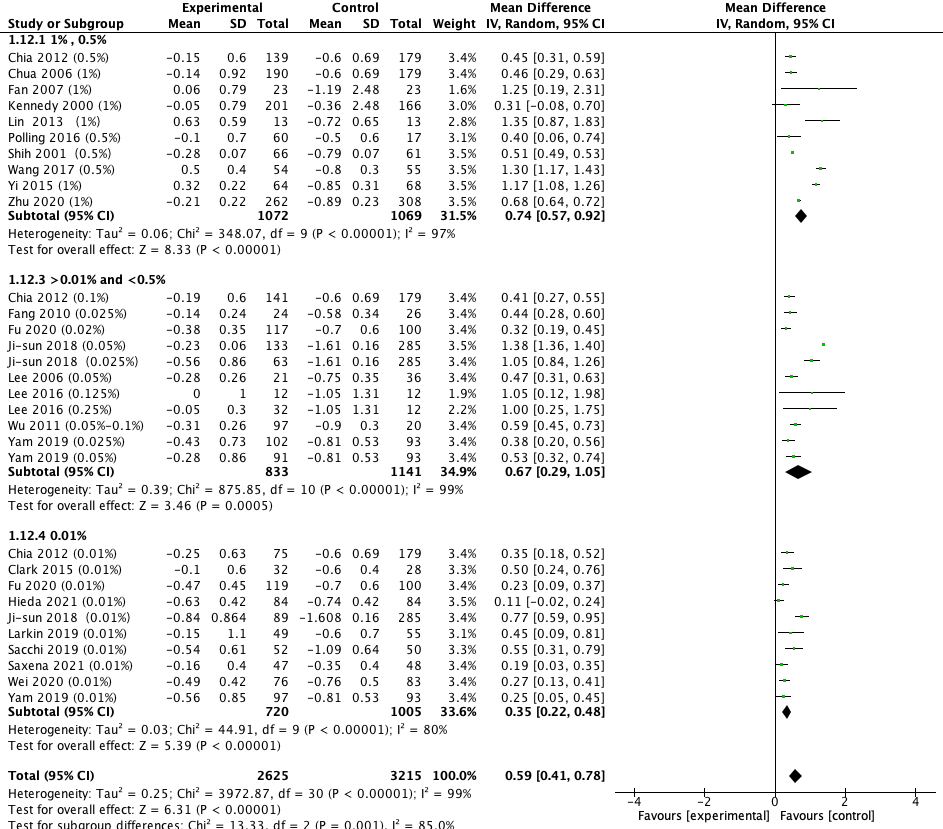
**

**eFigure 8B. Sensitivity Analysis after Removing Studies with Baseline Mean Refraction less than -4D**

**
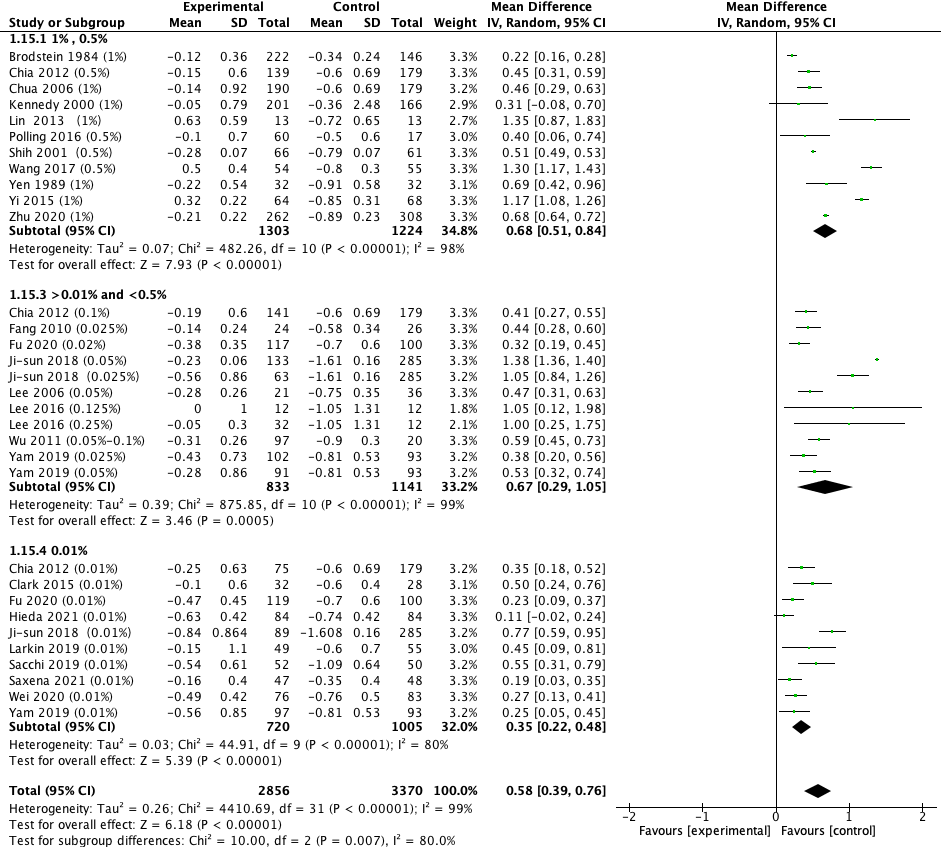
**

**eFigure 8C. Sensitivity Analysis after Removing Studies with a High Risk of Bias**

**
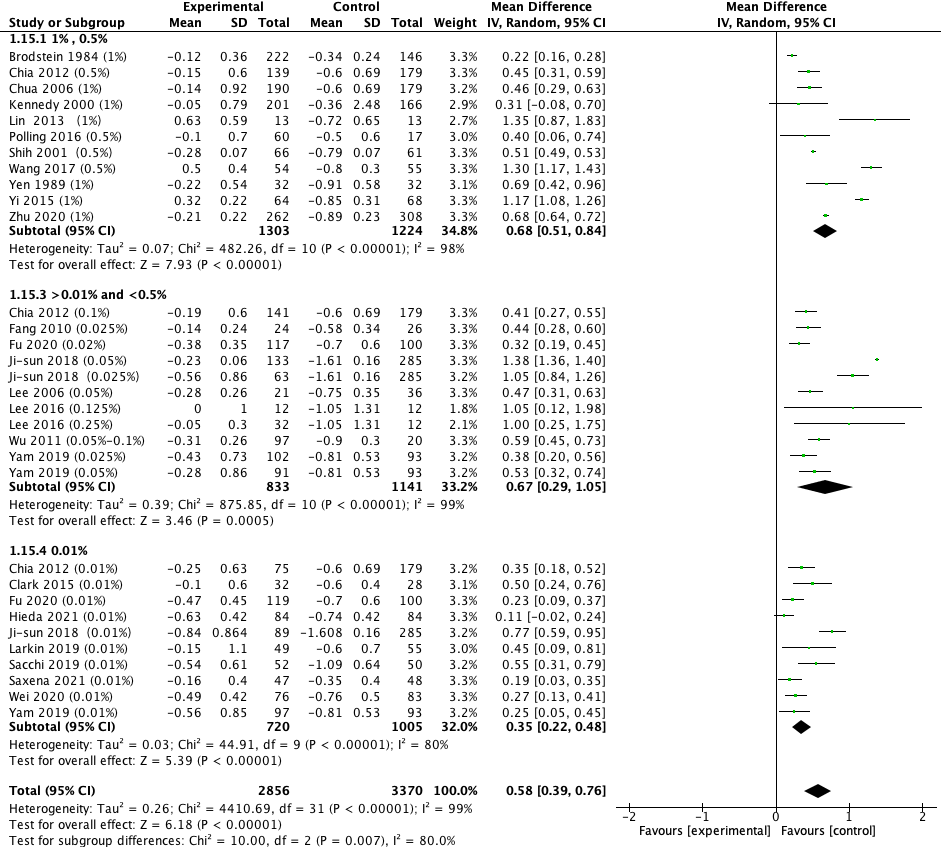
**

**eFigure 9. Funnel Plots for Publication Bias**

**A. B.**

**
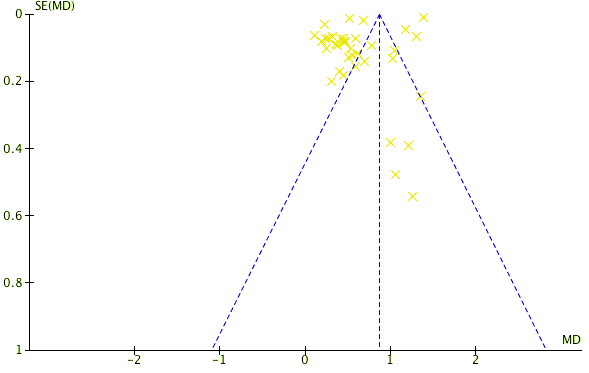

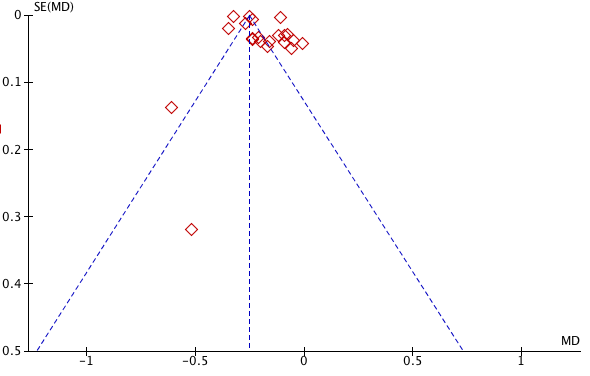
**

**C. D.**

**
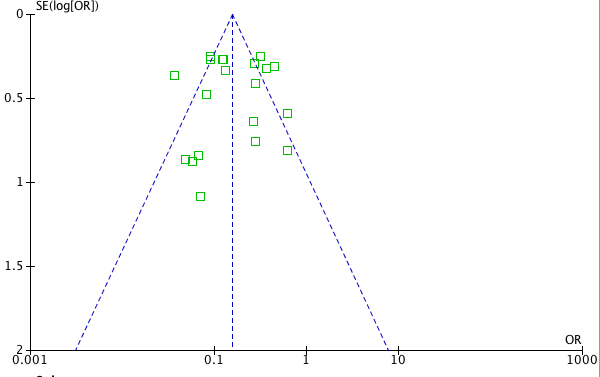

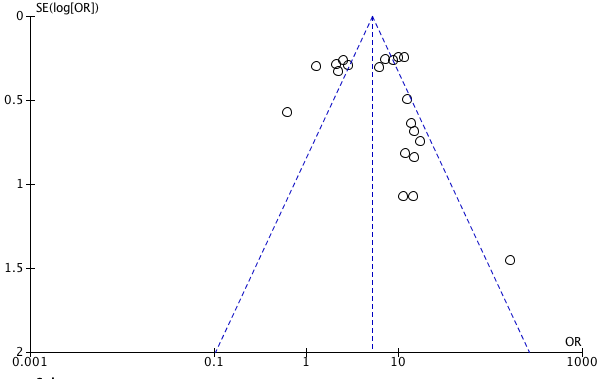
**

**E. F. G.**

**
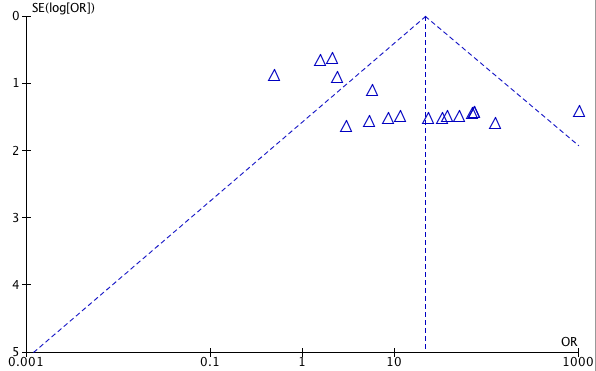

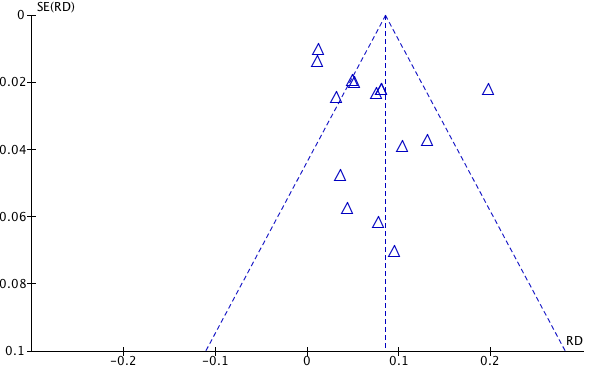

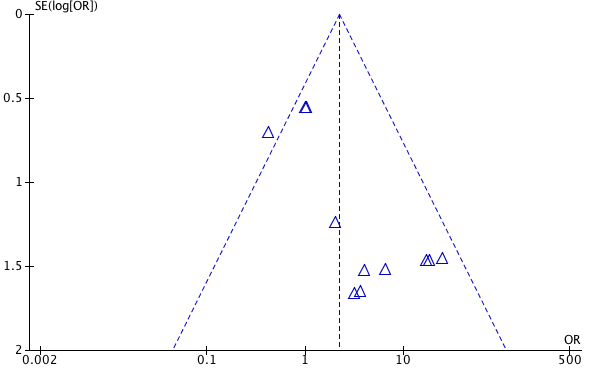
**

**Funnel plots evaluating publication bias for different outcomes: (A) mean annual refraction change, (B) mean annual axial  length change, (C) the number of children with rapid myopia progression, (D) the number of children with slow myopia progression, (E) photophobia, (F) blurred near vision and (G) allergy.**

**Reference**

1. Luu CD, Lau AM, Koh AH, Tan D. Multifocal electroretinogram in children on atropine treatment for myopia. *Br J Ophthalmol* 2005; **89**(2)**:** 151-153.

2. Liang CK, Ho TY, Li TC, Hsu WM, Li TM, Lee YC *et al.* A combined therapy using stimulating auricular acupoints enhances lower-level atropine eyedrops when used for myopia control in school-aged children evaluated by a pilot randomized controlled clinical trial. *Complementary therapies in medicine* 2008; **16**(6)**:** 305-310.

3. Chia A, Chua WH, Tan D. Effect of topical atropine on astigmatism. *The British journal of ophthalmology* 2009; **93**(6)**:** 799-802.

4. Tong L, Huang XL, Koh AL, Zhang X, Tan DT, Chua WH. Atropine for the treatment of childhood myopia: effect on myopia progression after cessation of atropine. *Ophthalmology* 2009; **116**(3)**:** 572-579.

5. Cooper J, Eisenberg N, Schulman E, Wang FM. Maximum atropine dose without clinical signs or symptoms. *Optometry and vision science : official publication of the American Academy of Optometry* 2013; **90**(12)**:** 1467-1472.

6. Chia A, Li W, Tan D, Luu CD. Full-field electroretinogram findings in children in the atropine treatment for myopia (ATOM2) study. *Documenta Ophthalmologica* 2013; **126**(3)**:** 177-186.

7. Kumaran A, Htoon HM, Tan D, Chia A. Analysis of changes in refraction and biometry of atropine- and placebo-treated eyes. *Investigative Ophthalmology and Visual Science* 2015; **56**(9)**:** 5650-5655.

8. Loughman J, Flitcroft D. The acceptability and visual impact of 0.01% atropine in a Caucasian population. *British Journal of Ophthalmology* 2016; **100**(11)**:** 1525-1529.

9. Diaz-Llopis M, Pinazo-Duran MD. Superdiluted atropine at 0.01% reduces progression in children and adolescents. A 5 year study of safety and effectiveness. *Archivos de la Sociedad Espanola de Oftalmologia* 2018; **93**(4)**:** 182-185.

10. Kinoshita N, Konno Y, Hamada N, Kanda Y, Shimmura-Tomita M, Kakehashi A. Additive effects of orthokeratology and atropine 0.01% ophthalmic solution in slowing axial elongation in children with myopia: first year results. *Japanese Journal of Ophthalmology* 2018; **62**(5)**:** 544-553.

11. Kaymak H, Fricke A, Mauritz Y, Lowinger A, Klabe K, Breyer D *et al.* Short-term effects of low-concentration atropine eye drops on pupil size and accommodation in young adult subjects. *Graefe's Archive for Clinical and Experimental Ophthalmology* 2018.

12. Tan Q, Ng ALK, Cheng GPM, Woo VCP, Cho P. Combined Atropine with Orthokeratology for Myopia Control: Study Design and Preliminary Results. *Current eye research* 2019; **44**(6)**:** 671-678.

13. Joachimsen L, Bohringer D, Gross NJ, Reich M, Stifter J, Reinhard T *et al.* A Pilot Study on the Efficacy and Safety of 0.01% Atropine in German Schoolchildren with Progressive Myopia. *Ophthalmology and therapy* 2019; **8**(3)**:** 427-433.
